# Intracellular Peptide N‑Myristoylation for Cancer Cell Ferroptosis without Acquired Resistance

**DOI:** 10.1021/jacs.5c15621

**Published:** 2025-10-21

**Authors:** Qiuxin Zhang, Weiyi Tan, Isabela Ashton-Rickardt, William Lau, Linrui Zou, Bing Xu

**Affiliations:** Department of Chemistry, 427969 Brandeis University, 415 South Street, Waltham, Massachusetts 02454, United States

## Abstract

N-Myristoylation, a well-known protein lipidation process, has yet to be explored for in situ peptide lipidation. Here, we report intracellular peptide N-myristoylation for potently inhibiting cancer cells. A self-assembling d-peptide, Gbb-NBD (**1**), comprising an N-terminal glycine, a d-dibiphenylalanine backbone, and a C-terminal nitrobenzofurazan, formed nanospheres in aqueous solution and exhibited strong cytotoxicity against cancer cells (GI_50_ = 500 nM) while sparing neuronal cells. Live-cell imaging showed that **1** traversed the plasma membrane to the ER, Golgi and mitochondria. NMT inhibition, LC-MS of cell lysates, and click chemistry confirmed the N-myristoylation of **1**. Functional studies showed that blocking NMT activity or modifying the N-terminus suppressed cytotoxicity, establishing N-myristoylation as essential for activity. Mechanistically, immunoblotting, lipidomic profiling, and rescue assays demonstrated that myristoylated **1** disrupted lipid metabolism and induced ferroptotic cell death, notably without the emergence of acquired resistance. In contrast, premyristoylated **1** displayed poor uptake and weak activity, underscoring the importance of in situ lipidation for cellular entry and function. Together, these findings reveal intracellular N-myristoylation of a short peptide as a new approach to drive ferroptosis and highlight its potential for developing membrane-targeting supramolecular therapeutics.

## Graphical abstract

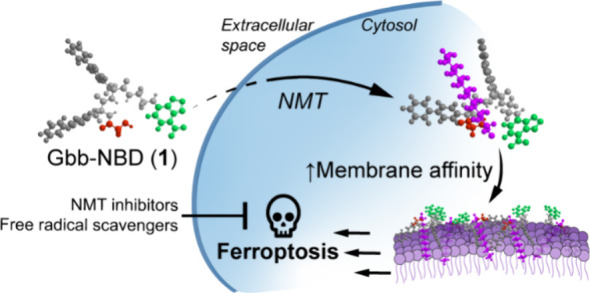

This communication reports the first example of intracellular peptide N-myristoylation for generating peptide assemblies to induce ferroptosis in cancer cells. Lipidation, a common posttranslational modification of proteins, plays key roles in cellular function and diseases.^1,2^ Among lipidation types, N-myristoylation, catalyzed by NMT,^3,4^ involves attaching myristic acid to N-terminal glycine residues of proteins.^5^ This modification governs membrane association, protein interactions, and localization, regulating processes from viral infections to oncogenesis.^6,7^ For instance, N-myristoylation aids viral capsid assembly, ensuring infectivity,^8,9^ and directs the membrane targeting of HIV proteins, crucial to the viral life cycle.^10,11^ In cancer, myristoylation affects protein stability, localization, and signaling critical to tumor progression. For example, it is essential for the oncogenic signaling of Src family kinases.^12^ Elevated NMT activity correlates with colorectal cancer, making it a potential biomarker and therapeutic target.^13,14^ It also enhances the lysosomal localization of LAMTOR1, promoting bladder cancer progression.^15^ Recent studies show that N-myristoylation of FSP1 drives phase separation,^16^ recruits FSP1 to the plasma membrane,^17^ and prevents ferroptosis.^18^ These findings underscore the multifaceted roles of myristoylation and have inspired the development of myristoylation inhibitors, showing promising preclinical efficacy.^19,20^

Advances in understanding protein myristoylation have spurred the exploration of chemical and chemoenzymatic myristoylation.^21–23^ Researchers have developed lipidation and ligation chemistry for synthesizing and semisynthesizing homogeneous lipidated proteins.^24,25^ Solid-phase myristoylation methods are efficient.^26^ Synthetic myristoylation of peptides forms monolayers,^27^ improves the pharmacokinetics of ELPs for drug delivery,^28^ enhances the antibacterial activity of defensins,^29^ and enables the production of protein amphiphiles in *E. coli* for biomaterial design.^30^ Myristoylation of ω-conotoxin MVIIA increases its therapeutic efficacy while reducing side effects, demonstrating its potential for optimizing peptide-based drugs.^31^ A recent study reports a method for selective pull-down assays of N-terminal glycine peptides from mixtures without prior knowledge of peptide distribution.^32^

Despite these advances, intracellular peptide N-myristoylation, the use of cellular machinery (e.g., NMT) to myristoylate synthetic peptides in situ, remains unachieved. Several challenges impede progress: Most synthetic peptides contain L-amino acids and have limited proteolytic stability in vivo, making N-myristoylation unlikely before proteolysis or unable to maintain integrity after lipidation. Although NMT is present in both the cytoplasm and membranes, myristoylation likely happens near membranes due to the hydrophobic myristoyl group. Finally, no facile assay exists to monitor peptide myristoylation in cells. Thus, a general approach that enables peptide N-myristoylation, reports this modification in cells, and assesses its effects would be valuable.

We attached glycine to the N-terminus of bb-NBD, generating Gbb-NBD (**1**) as an NMT substrate. **1** formed nanospheres in aqueous solution, potently inhibited the growth of cancer cells (GI_50_ ∼ 500 nM), and showed minimal neuronal toxicity. Live-cell imaging revealed rapid translocation from the plasma membrane to the ER, Golgi and mitochondria within 5 min. NMT inhibition, LC-MS, and click chemistry, confirmed intracellular N-myristoylation of **1**. Lip-1 ^33,34^ rescued cells treated with **1**, and lipidomics showed elevated ferroptotic lipids, together implicating ferroptosis as the main death pathway. Consistent with its self-assembly, **1** induced ferroptosis without acquired resistance. The premyristoylated analog **2** demonstrated that in situ lipidation is critical for activity; N-terminal acetylation (**3**) abolished cytotoxicity, confirming the need for N-myristoylation. The phenylalanine-based analog Gff-NBD (**4**) exhibited poor membrane uptake and low cytotoxicity, underscoring the role of the dibiphenylalanine in membrane interactions. These findings establish a strategy for intracellular lipidation of membrane-affinitive small molecules, enabling supramolecular assemblies that target the endomembrane system of cancer cells.

We recently identified a dipeptide containing b, bb-NBD, which displaced cholesterol from membrane,^35^ indicating strong membrane affinity. The built-in NBD fluorophore enabled direct, real-time imaging of bb-NBD uptake and distribution. Incorporating b into a rigid-rod aromatic structure^36^ also accelerated cellular uptake.^37^ Thus, we used the bb motif to target endomembrane system and access cytosolic enzymes such as NMT. Accordingly, we introduced an N-terminal glycine to bb-NBD to generate Gbb-NBD (**1**) (Scheme 1). We next synthesized an N-myristoylated analog (myr-Gbb-NBD (**2**)) and an N-acetylated analog (**3**) to assess the role of lipidation. To evaluate the contribution of the bb motif to membrane anchoring, we also prepared the phenylalanine-based analog, Gff-NBD (**4**).

**Scheme 1:**
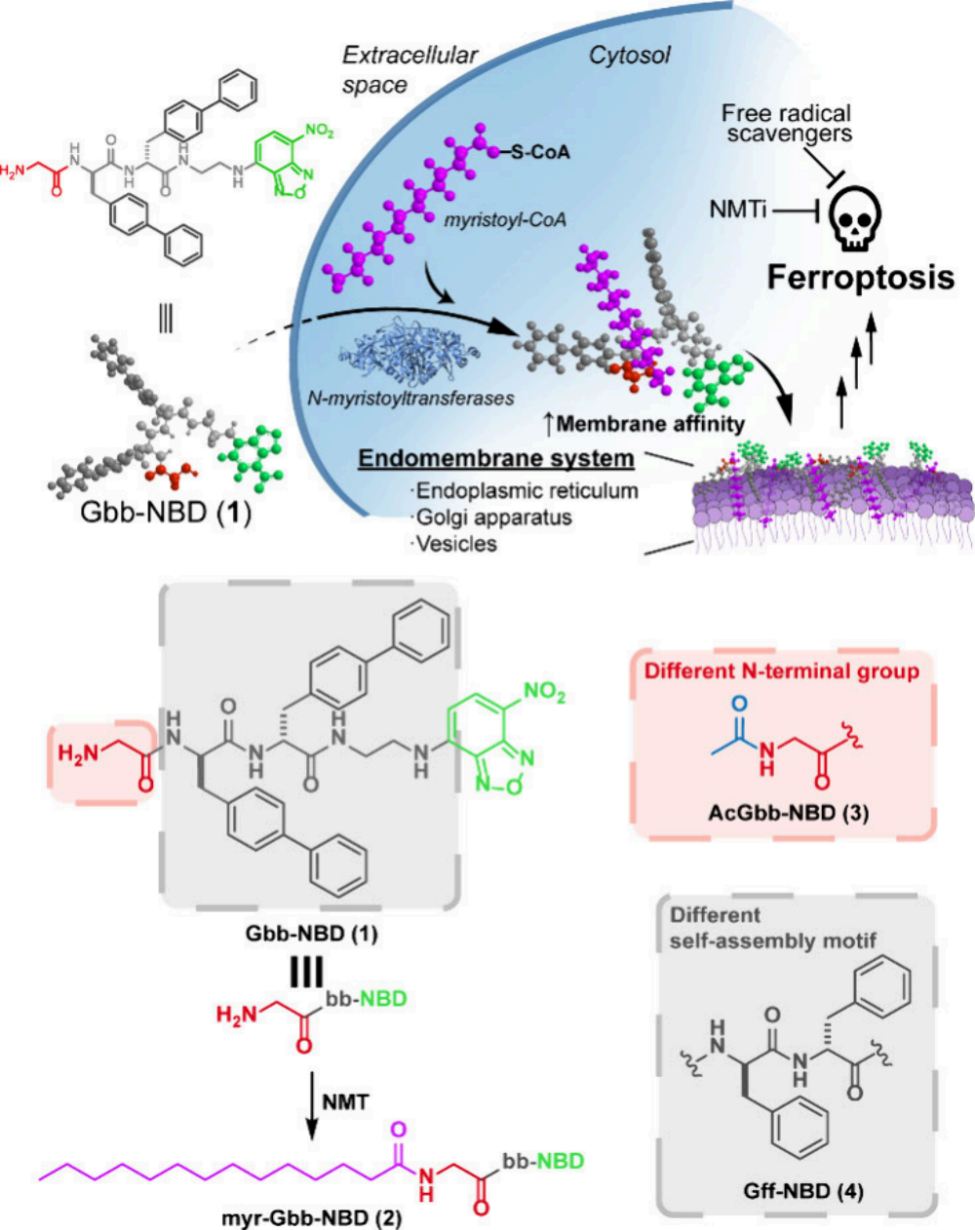
Illustration of Intracellular Myristoylation of Peptide **1** Inducing Ferroptosis

After synthesizing **1**-**4** (Schemes S1–S2, Figures S1–S5), we found that **1** exhibited a critical aggregation concentration (CAC) of 9.9 μM and self-assembled into nanospheres in water (Figures S6–S7). We evaluated its cytotoxicity against HeLa and SH-SY5Y cells (Figures 1A, S8–S11), chosen for their distinct NMT expression levels (Figure S12) and broad use as models in cancer biology and neuroscience, respectively. MTT assays showed that **1** potently inhibited HeLa growth (24 h GI_50_ = 0.5 μM), over 10-fold lower than in SH-SY5Y cells (Figure 1B). The viability–concentration curve showed a biphasic dose–response with inflection points near CAC of **1** and its myristoylated analog **2** (Figures 1A, S13), suggesting that myristoylation enhances potency against HeLa cells.

**Figure 1:**
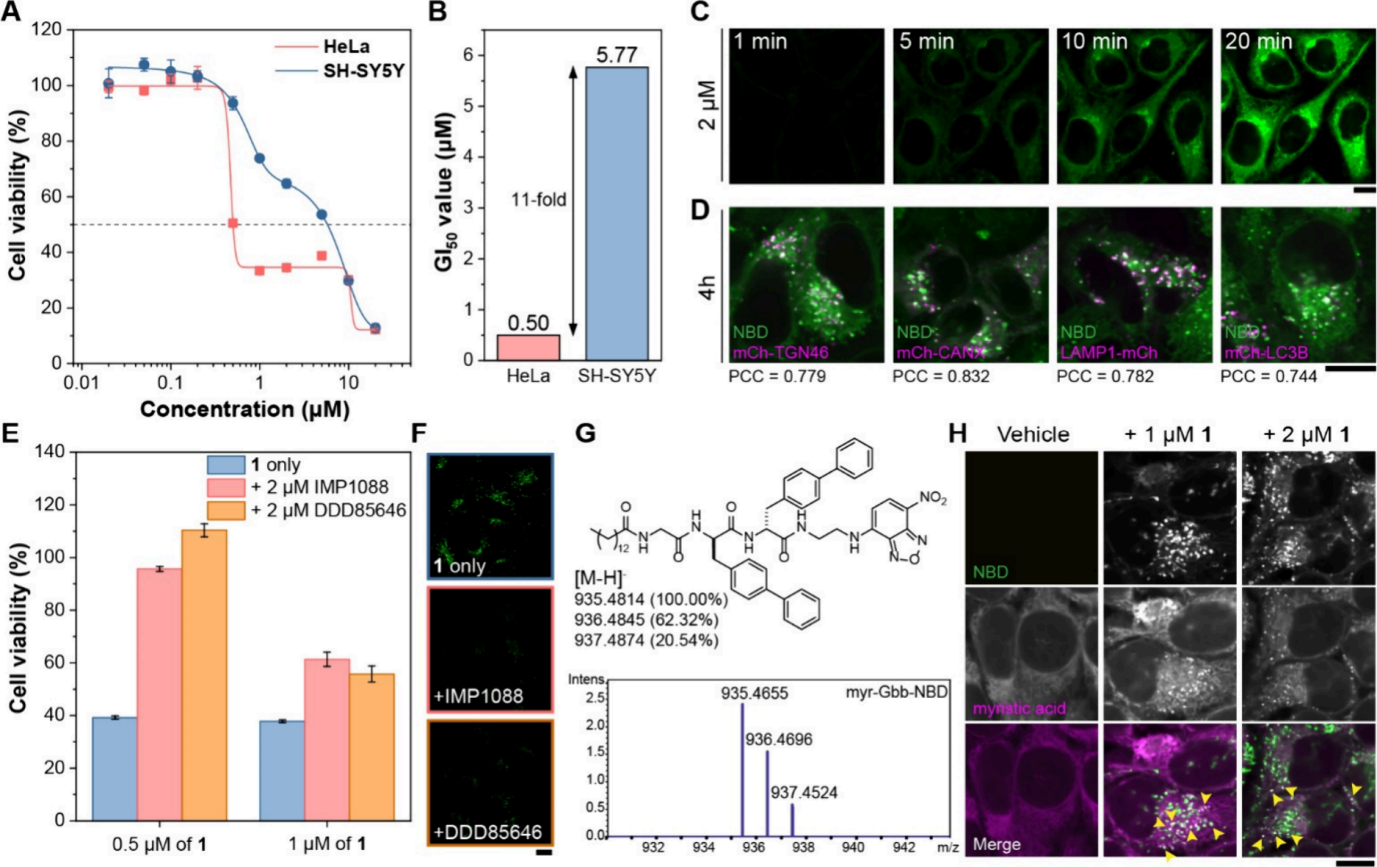
(A) Cell viability and (B) GI_50_ values of HeLa and SH-SY5Y cells treated with **1** for 24h. (C) Time-lapse and (D) CLSM images of HeLa cells treated with 2 μM of **1**. (E) Cell viability and (F) CLSM images of HeLa cells treated with 0.5 μM **1** with or without NMT inhibitors (IMP1088, DDD85646) for 24h. (G) HRMS spectrum of myr-Gbb-NBD from lysate of HeLa cells treated with **1**. (H) CLSM images of HeLa cells tracked for myristic acid using click chemistry. Yellow arrowheads denote colocalization. Scale bar = 10 μm.

Time-lapse CLSM revealed membrane association immediately upon addition of **1**, followed by translocation to the ER, Golgi and mitochondria within 5 min (Figures 1C, S14–S15). After 4 h, fluorescence concentrated in puncta that colocalized with the TGN (mCh-TGN46), endoplasmic reticulum (mCh-CANX), lysosomes (LAMP1-mCh), autophagosomes (LC3B-mCh), and lipid droplets (mCh-HPos) (Figures 1D, S16–S25), indicating preferential targeting of endomembrane organelles central to intracellular trafficking, degradation, and lipid metabolism. The initial colocalization with mitochondria (mCh-TOMM20) gradually diminished, with the weak residual NBD signal likely reflecting nonspecific interactions (Figure S26).

We next confirmed intracellular myristoylation using multiple methods. Co-treatment with two potent NMT inhibitors, IMP1088 ^38^ and DDD85646,^39^ significantly rescued cells from **1**-induced cytotoxicity without notable toxicity at the tested concentrations (Figures 1E, S27). CLSM imaging further revealed a marked reduction of intracellular fluorescent puncta upon NMT inhibition, consistent with the requirement for NMT-instructed intracellular self-assembly for cytotoxicity (Figures 1F, S28–S31). At higher concentrations of both Gbb-NBD and NMT inhibitors, synergistic effects were observed, producing more cellular aggregates, possibly due to disrupted lipidation homeostasis and impaired intracellular trafficking. Time-lapse CLSM images showed reduced internalization and accumulation of **1** when coincubated with NMT inhibitors (Figures S32–S33). Moreover, SH-SY5Y cells exhibited markedly lower uptake of **1** compared to HeLa cells, correlating with their reduced susceptibility (Figures S34–S35). Triacsin C,^40^ an inhibitor of acyl-CoA synthetases, significantly rescued cells from **1** by depleting intracellular myristoyl-CoA at cell-compatible concentrations, confirming the essential role of myristoylation (Figure S36). HRMS identified myristoylated **1** in lysates from treated but not untreated cells (Figure 1G). Although unmodified **1** remained detectable, even partial lipidation sufficed to induce cytotoxicity. Finally, a bioorthogonal click reaction^41,42^ between azido myristic acid and Cy5 alkyne in cells produced puncta that strongly colocalized with **1**, confirming consumption of myristic acid during the formation of N-myristoylated peptide assemblies (Figures 1H, S37, Scheme S3).

Given the potent cytotoxicity arising from the in situ myristoylation of **1**, we examined the cell death pathway in HeLa cells. The ferroptosis inhibitor Lip-1 rescued **1**-induced cytotoxicity in a dose-dependent manner (Figures 2A, S38), indicating a ferroptotic mechanism, and showed no toxicity at the tested concentrations (Figure S38C). CLSM showed reduced intracellular fluorescence from **1** in Lip-1-treated cells, consistent with ferroptosis suppression (Figures 2B, S39–S40). The weaker radical scavenger Fer-1 ^34^ provided partial rescue (Figures S38, S41–S42), whereas the iron chelator DFO was ineffective (Figure S43), supporting a lipid peroxidation–dependent cell death. Immunoblotting showed decreased levels of the ferroptosis suppressor GPX4, while ACSL4 remained largely unchanged. At 5 μM, GPX4 partially recovered, likely reflecting crosstalk with alternative death pathways. Concurrently, the LC3B–II/LC3B–I ratio increased with dose (Figure 2C). These changes indicate a ferroptotic pathway primarily driven by impaired detoxification of lipid peroxides due to GPX4 depletion, with autophagy engagement as a plausible contributor. While lipid ROS accumulation appears central to this process, other contributing factors cannot be excluded. In contrast, inhibitors of other canonical cell death pathways failed to mitigate **1**-induced cytotoxicity (Figure S44), confirming ferroptosis as the predominant mechanism.

**Figure 2:**
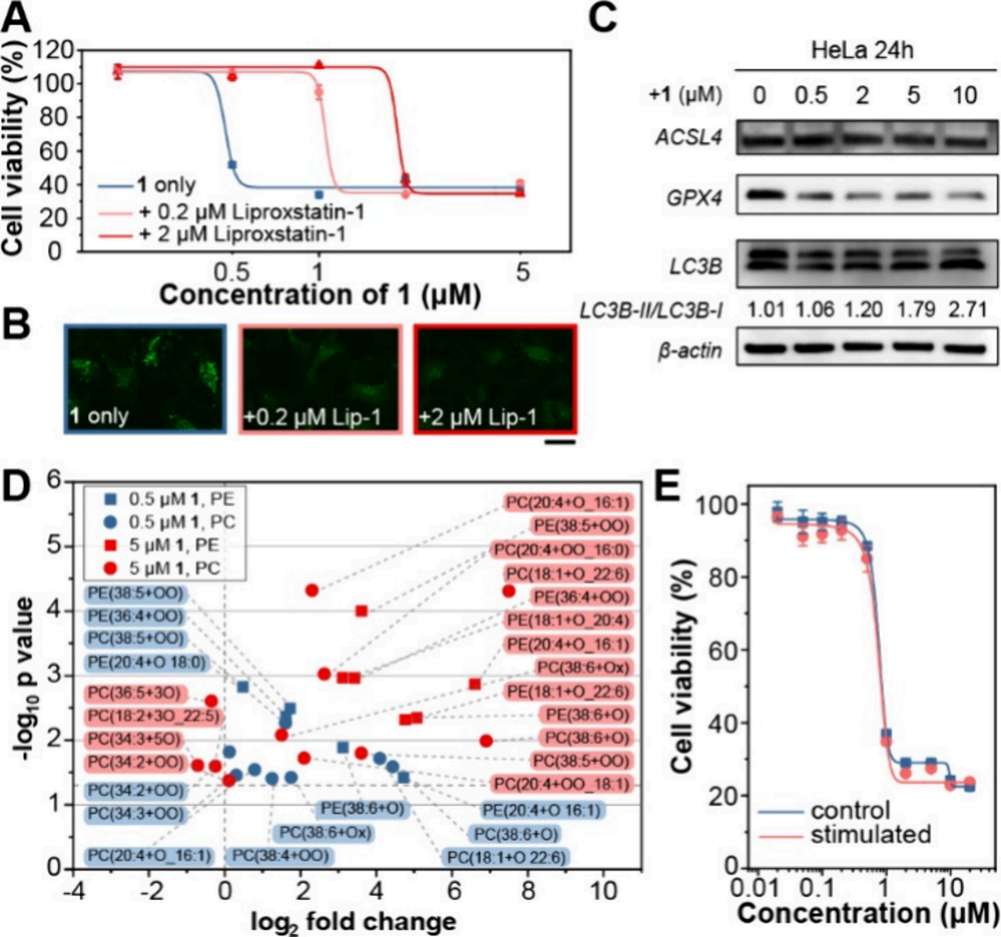
(A) Cell viability and (B) CLSM images of HeLa cells treated with **1** with or without liproxstatin-1 for 24 h. (C) Immunoblotting of ferroptosis/autophagy related proteins in HeLa cells with or without the treatement of **1**. (D) Quantitative lipodomics of ferroptosis marker lipids in HeLa cells treated with **1** for 24h. (E) Cell viability of control or stimulated HeLa cells treated with **1** for 24 h. Scale bar = 20 μm.

Untargeted lipidomics further corroborated the ferroptotic mechanism, showing significant accumulation of oxidized PEs and PCs at 0.5 μM of **1**, which intensified at 5 μM (Figure 2D, S45), hallmarking lipid-peroxidation during ferroptosis. Collectively, these data demonstrate that in situ myristoylation of **1** triggered ferroptotic cell death in HeLa cells. Following an established protocol,^43^ cells that survived escalating concentrations of **1** were designated as the stimulated group. Comparison of **1**-induced cytotoxicity between the stimulated and control groups revealed no significant differences (Figure 2E), indicating that **1** hardly elicits acquired resistance.

To test whether in situ myristoylation is essential for activity, we synthesized **2**, the premyristoylated **1**. **2** also self-assembled into nanospheres, as revealed by TEM (Figure S46), above its CAC of 0.32 μM (Figure S13). MTT assays showed markedly reduced cytotoxicity (Figure 3A), with GI_50_ > 20 μM (Figure 3C). CLSM from the same batch revealed significantly lower cellular uptake of **2** than **1** (Figures 3B, S47–S50), and prolonged treatment of **2** did not induce intracellular aggregation (Figure S51). Together, these findings indicate that preinstalled lipidation hinders membrane permeation and cellular uptake due to the lack of an ionizable group and the lower CAC of **2**, underscoring that in situ myristoylation within cells is crucial for the bioactivity of **1**.

**Figure 3:**
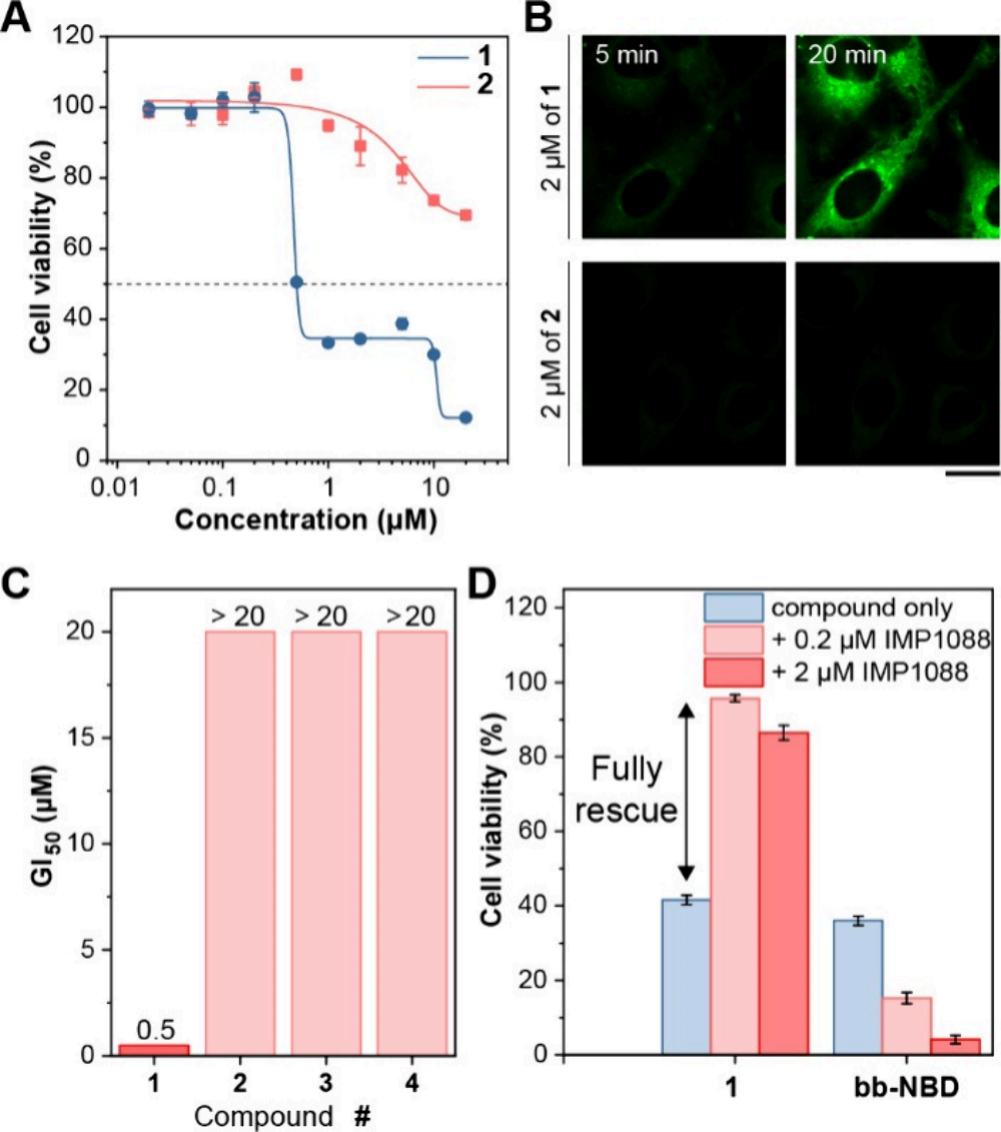
(A) Cell viability of HeLa cells treated with **1** or **2** for 24h. (B) Time-lapse CLSM images of HeLa cells treated with 2 μM of **1** or **2**. (C) Summary of GI_50_ values of **1** through **4** for 24 h. (D) Cell viability of HeLa cells treated with **1** (0.5 μM) or bb-NBD (20 μM) with or without IMP1088 for 24 h. Scale bar = 20 μm.

Two additional controls, **3** (N-acetylated) and **4** (bearing the ff motif), also showed significantly reduced cytotoxicity, (GI_50_ > 20 μM; Figures 3C, S52–S53) and decreased cellular uptake (Figures S54–S58), demonstrating the requirement of a free amine for lipidation and the bb motif for membrane targeting. Finally, IMP1088 was unable to rescue cells treated by bb-NBD^35^ (Figures 3D, S59), which lacks an N-terminal glycine, confirming that an N-terminal glycine is essential for the myristoylation and activity of **1**.

In conclusion, this work presents a strategy for generating intracellular assemblies that trigger ferroptosis in cancer cells while mitigating acquired drug resistance, complementing existing inhibitor- and degrader-based approaches.^44^ It expands the scope of EISA^45^ from catalyzing bond-breaking^46–57^ and bond formation^58–60^ reactions to utilizing endogenous lipidation machinery, an underexplored routing for cancer targeting. Notably, while NMT upregulation can confer resistance to inhibitors, it would instead boost the production of lipidated peptides that suppress cancer cells, suggesting therapeutic potential for this mechanism. Lipidomics data reveal a concentration-dependent action: lower concentrations primarily induce ferroptosis, while higher ones engage additional death pathways, including apoptosis and necroptosis (Figure S60), consistent with the biphasic dose–response (Figure 1A). While proteins can adopt myr-exposed or myr-sequestered conformations,^61^ further study is needed to elucidate the conformation of myristoylated peptide **1**. Beyond therapy, the ability to anchor functionalized peptides onto lipid membranes offers a versatile platform for probing membrane dynamics and tailoring extracellular vesicles. While additional investigations are required to clarify mechanism and quantify the extent of myristoylation, these findings suggest a new framework for lipidation-driven self-assembly with broad biomedical implications.

## Supplementary Material



## Notes

Materials and detailed experimental procedures, chemical structures and characterization of compounds, TEM and CLSM images, cell viabilities (PDF)

## Abbreviations

ACSL4: acyl-CoA synthetase long-chain family member 4
b: D-biphenylalanine
CAC: critical aggregation concentration
CLSM: confocal laser scanning microscopy
CoA: coenzyme A
DFO: deferoxamine
ELPs: elastin-like polypeptides
ER: endoplasmic reticulum
EISA: enzyme-instructed self-assembly
f: d-phenylalanine
Fer-1: ferrostatin-1
FSP1: ferroptosis suppressor protein 1
GPX4: glutathione peroxidase 4
HIV: human immunodeficiency virus
HRMS: high-resolution mass spectrometry
LAMTOR1: late endosomal/lysosomal adaptor, MAPK and mTOR activator 1
LAMP1-mCh: lysosomal-associated membrane protein 1-mCherry
mCh-LC3B: mCherry-microtubule-associated protein 1 light chain 3 β
LC-MS: liquid chromatography–mass spectrometry
Lip-1: liproxstatin-1
mCh-CANX: mCherry-calnexin
mCh-HPos: pEFIRES-P-mCherry-HPos
mCh-TGN46: mCherry-trans-Golgi network glycoprotein 46
mCh-TOMM20: mCherry-translocase of the outer mitochondrial membrane 20
MVIIA: ω-conotoxin MVIIA
NBD: nitrobenzofurazan
MTT: 3-(4,5-dimethylthiazol-2-yl)-2,5-diphenyltetrazolium bromide
NMT: N-myristoyltransferase
PCs: phosphatidylcholines
PEs: phosphatidylethanolamines
ROS: reactive oxygen species
TGN: trans-Golgi network.
