# Supplementary material for: Intracellular Peptide N‑Myristoylation for Cancer Cell Ferroptosis without Acquired Resistance
Source: J Am Chem Soc. 2025 Oct 21;147(44):40120–5. doi: 10.1021/jacs.5c15621 (PMC12593337; doi:10.1021/jacs.5c15621)
Supplement: Supplementary file 1 [file ja5c15621_si_001.pdf]

Supporting Information for

Intracellular Peptide N-Myristoylation for Cancer Cell Ferroptosis without Acquired Resistance

Qiuxin Zhang, Weiyi Tan, Isabela Ashton-Rickardt, William Lau, Linrui Zou and Bing Xu*

Department of Chemistry, Brandeis University, 415 South Street, Waltham, MA 02454, USA

Contents

S1. Experimental materials and instruments 2

S2. Synthesis and characterization of compounds 3

S3. Critical aggregation concentration (CAC) determination 12

S4. TEM sample preparation 12

S5. Cell culture 12

S6. MTT assays 13

S7. Confocal laser scanning microscopy (CLSM) imaging 13

S8. Cell click reaction 14

S9. Plasmid transfection 15

S10. Detection of peptide myristoylation by HR-MS 15

S11. Drug resistance test 16

S12. Untargeted lipidomics profiling 17

S13. Supplemental figures 18

S14. Reference 59

S1. Experimental materials and instruments

Materials

2-Cl trityl chloride resin (1.0-1.2 mmol/g), Fmoc-D-phe-OH and HBTU were purchased from GL Biochem (Shanghai, China). Acetyl anhydride was purchased from Sigma-Aldrich. Fmoc-D-4,4'-biphenylalanine was purchased from ChemScene. DIEA, 4-chloro-7-nitrobenzofurazan, myristic acid and solvents were purchased from Fisher Scientific. Azido myristic acid and Click-&-Go® Cell Reaction Buffer Kit was purchased from Vector Laboratories. Cy5 alkyne was purchased from Lumiprobe. IMP1088, DDD85646, Liproxstatin-1 were purchased from MedChemExpress. All the chemical reagents and solvents were used as received from commercial sources without further purification.

ACSL4 Rabbit monoclonal antibody (Catalog# 38493) and GPX4 antibody (Catalog# 52455) were purchased from Cell Signaling Technology. Rabbit recombinant monoclonal LC3B antibody (Catalog# ab192890) and anti-β actin antibody (Catalog# ab8227) were purchased from Abcam. Goat anti-rabbit IgG (H+L) secondary antibody, HRP (Catalog# 31460) was purchased from Invitrogen. Hoechst 33342 was purchased from Invitrogen. CellMask™ Plasma Membrane Deep Red was purchased from Invitrogen.

mCherry-TGNP-N-10 was a gift from Michael Davidson lab (Addgene plasmid #55145). mCherry-Calnexin-N-14 was a gift from Michael Davidson lab (Addgene plasmid #55005). pLAMP1-mCherry was a gift from Amy Palmer lab (Addgene plasmid #45147). mCherry-hLC3B-pcDNA3.1 was a gift from David Rubinsztein lab (Addgene plasmid #40827). pEFIRES-P-mCherry-HPos was a gift from Elina Ikonen lab (Addgene plasmid #87159).

mCherry-TOMM20-N-10 was a gift from Michael Davidson lab (Addgene plasmid #55146). Xfect™ Transfection Reagent (Catalog # 631317) was purchased from TaKaRa.

Minimum Essential Media (MEM), Ham's F-12 Media (F-12), fetal bovine serum (FBS) and Penicillin-Streptomycin were purchased from Gibco by Life Technologies. Eagle's Minimum Essential Medium (EMEM) were purchased from ATCC.

Instruments

All compounds were purified by a reverse phase HPLC (Agilent 1100 Series) equipped with an Xterra C18 RP column. HPLC grade acetonitrile (0.1% TFA) and HPLC grade water (0.1% TFA) were used as eluents. The LC-MS spectra were obtained with a Bruker Elute PLUS UHPLC with a Bruker timsTOF Pro. Transmission electron microscopic images were obtained on Morgagni 268 transmission electron microscope. Fluorescent images were taken by Nikon AX-R Confocal System at the lens of 60× with oil.

S2. Synthesis and characterization of compounds

Synthesis of NBD-ethylenediamine

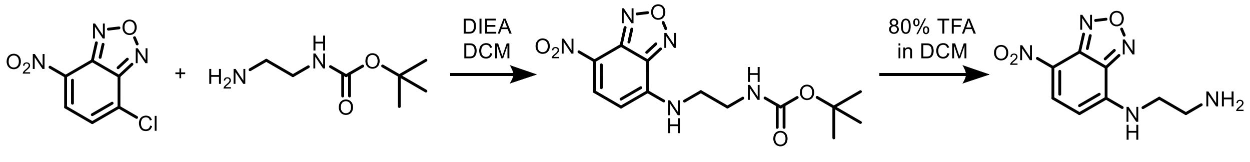

Scheme S1. Synthetic route of NBD-ethylenediamine

N-Boc-1,2-diaminoethane (1.2 equiv.) was dissolved in DCM and reacted with NBD-Cl (1 equiv.) at room temperature. DIEA was added dropwise to maintain the pH around 8. After stirring for 6 hours, the mixture was diluted with DCM, and the organic phase was sequentially washed with aqueous $NaHCO_3$ and water. The organic layer was then air-dried, and the resulting crude product was treated with 80% TFA in DCM for 1 h. Finally, the reaction mixture was evaporated to dryness, redissolved in H_2O/acetonitrile, and lyophilized.

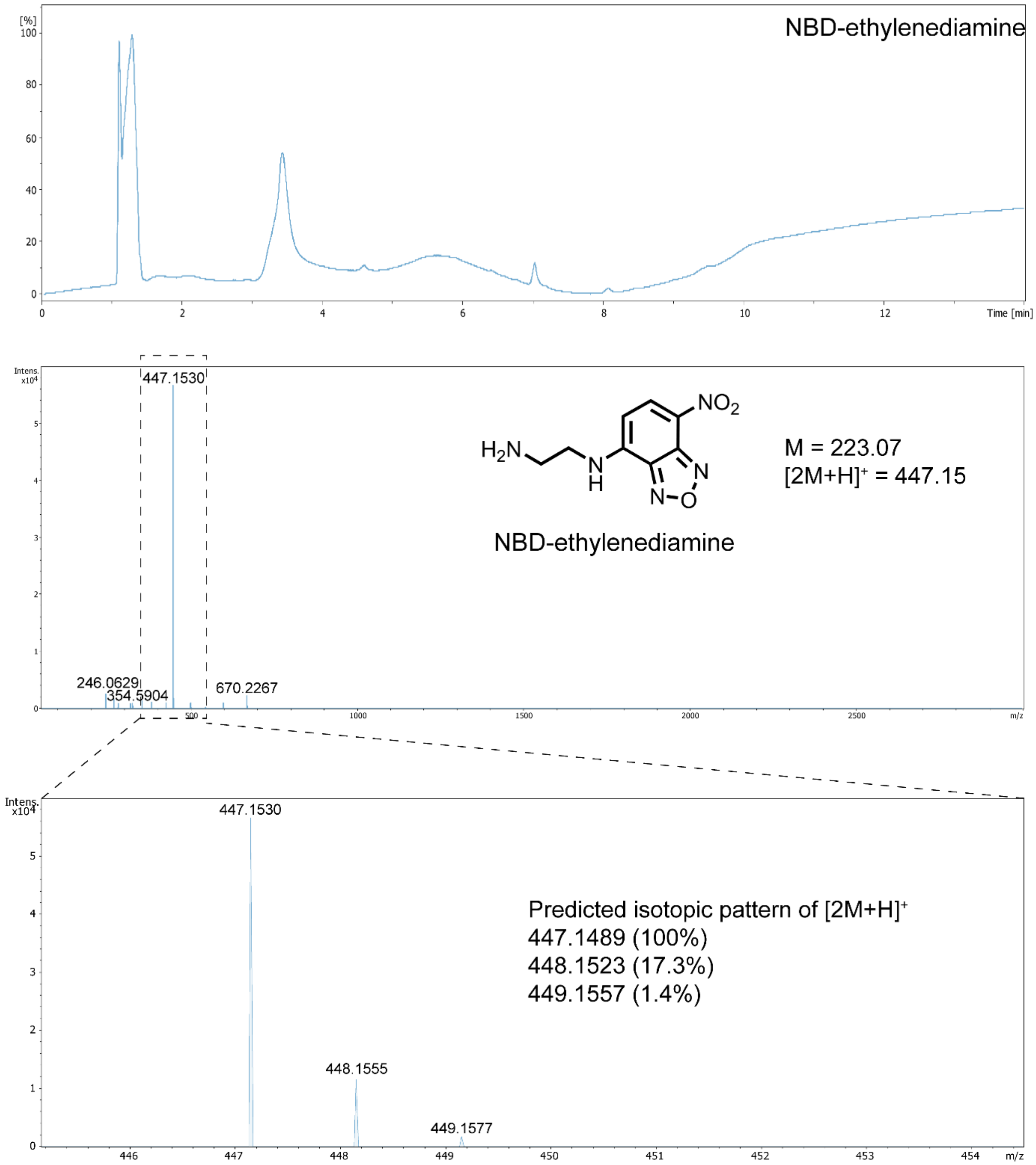

Figure S1. High resolution mass spectrum of NBD-ethylenediamine.

Solid phase peptide synthesis

Peptides were synthesized using standard Fmoc solid-phase peptide synthesis on 2-chlorotrityl chloride resin with the corresponding Fmoc-protected amino acids. The 2-Cl resin was first swollen in dry DCM for 30 min, after which the initial Fmoc-protected amino acid was loaded. Remaining active sites were capped with a solution of DCM:MeOH:DIEA (17:2:1) for 15 min. The Fmoc group was removed with 20% piperidine in DMF for 20 min, and the next Fmoc-protected amino acid was coupled using HBTU as the activating reagent. After completion of the synthesis, the peptide was cleaved from the resin with TFA/TIPS/H_2O (95:2.5:2.5) for 1 h. The crude peptide solution was air-dried, precipitated with diethyl ether, and centrifuged to obtain the solid, which was further dried by lyophilization. Lowercase letters denote D-amino acids.

Synthesis of peptides 2 and 3

After the removal of the Fmoc group from the second biphenylalanine, myristic acid (for **2**) and acetyl anhydride (for **3**) were coupled to its free amino group. After completion, the peptide chain was cleaved and collected as described in Solid Phase Peptide Synthesis for the next step.

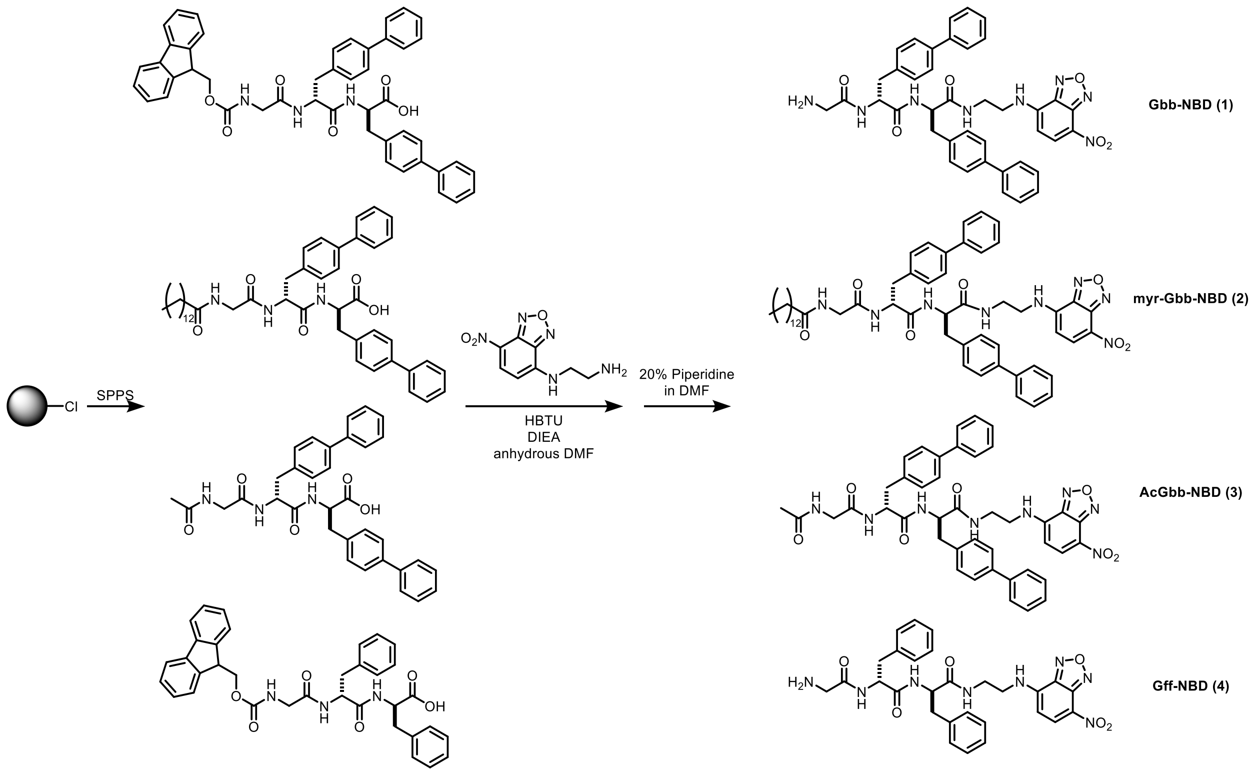

Scheme S2. Synthetic route of peptides 1-4.

Synthesis of peptides 1-4

In brief, 0.1 mmol of the synthesized peptide (Fmoc-bb for **1**, myr-bb for **2**, AcGbb for **3** and Fmoc-ff for **4**) was dissolved in 1 mL of dry DMF, and 0.12 mmol of HBTU was added directly to the solution. DIEA was introduced dropwise to adjust the pH to ~8. After stirring for 30 min, 0.12 mmol of NBD-ethylenediamine was added, with the pH maintained at 8 using DIEA. The mixture was stirred overnight, after which the solvent was evaporated. The Fmoc group was then removed with 20% piperidine for 20 min. Following another round of solvent removal, the crude product was dissolved in MeOH and purified by RP-HPLC to yield the desired compounds.

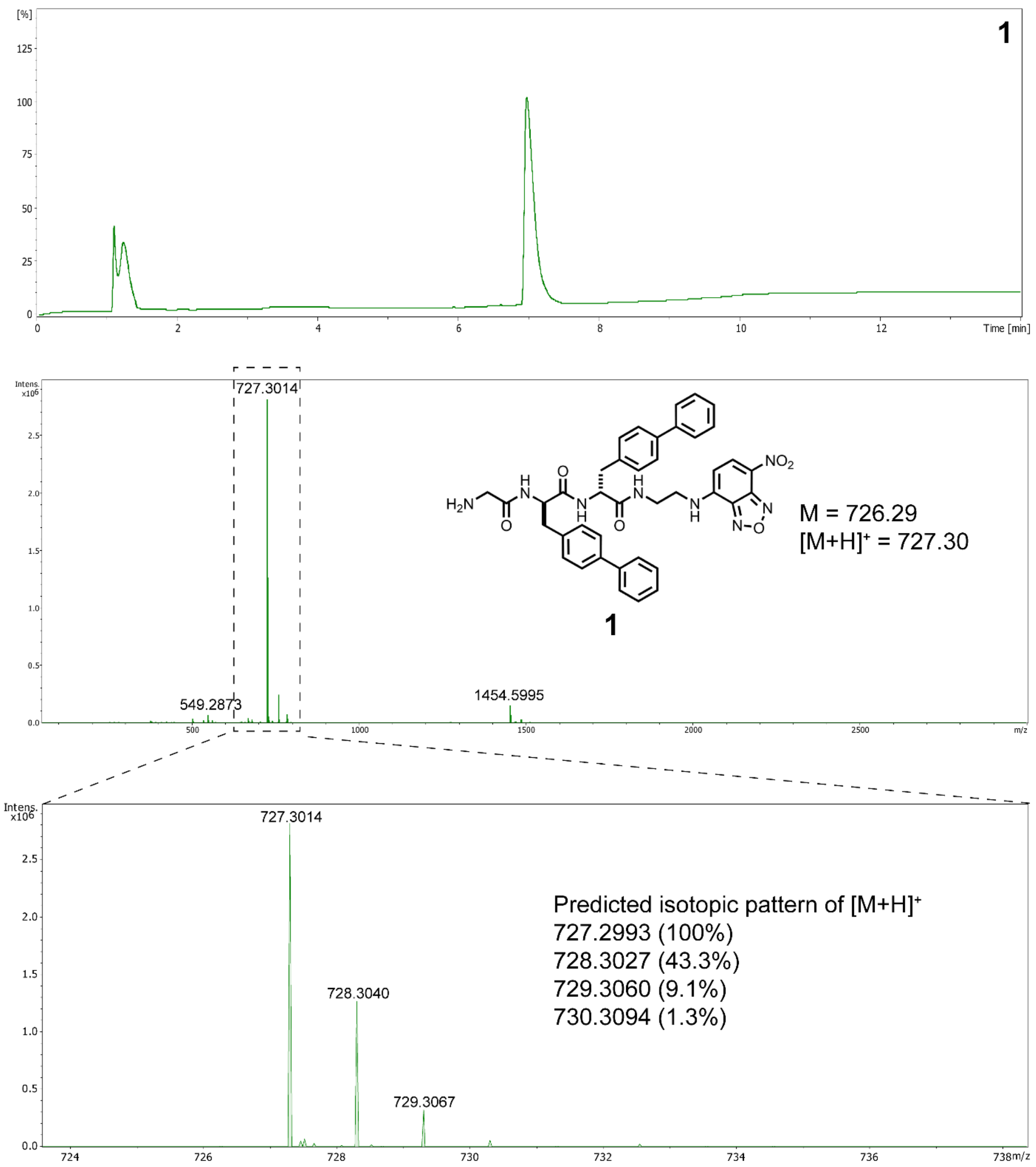

Figure S2. High resolution mass spectrum of Gbb-NBD (**1**).

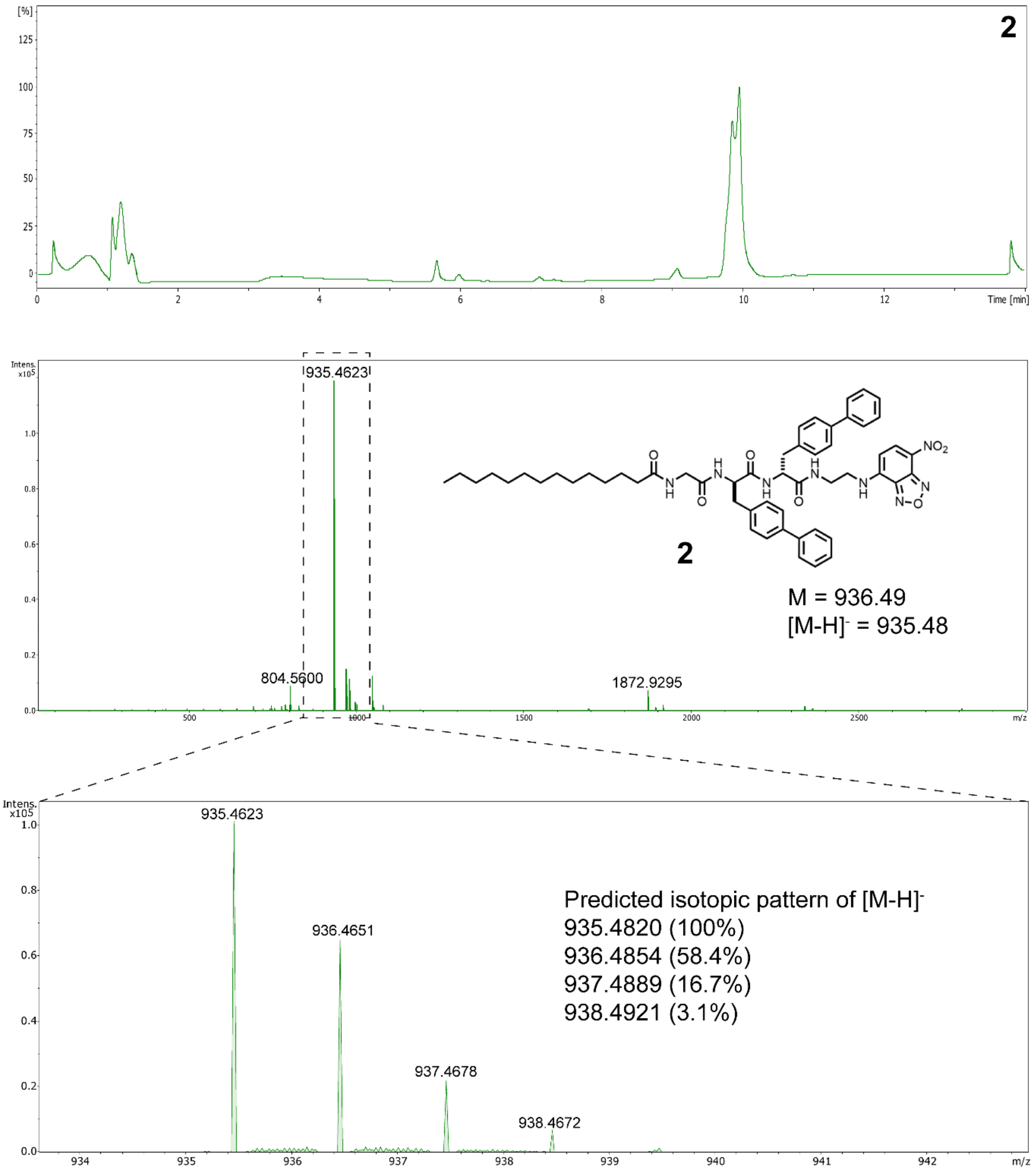

Figure S3. High resolution mass spectrum of myr-Gbb-NBD (**2**).

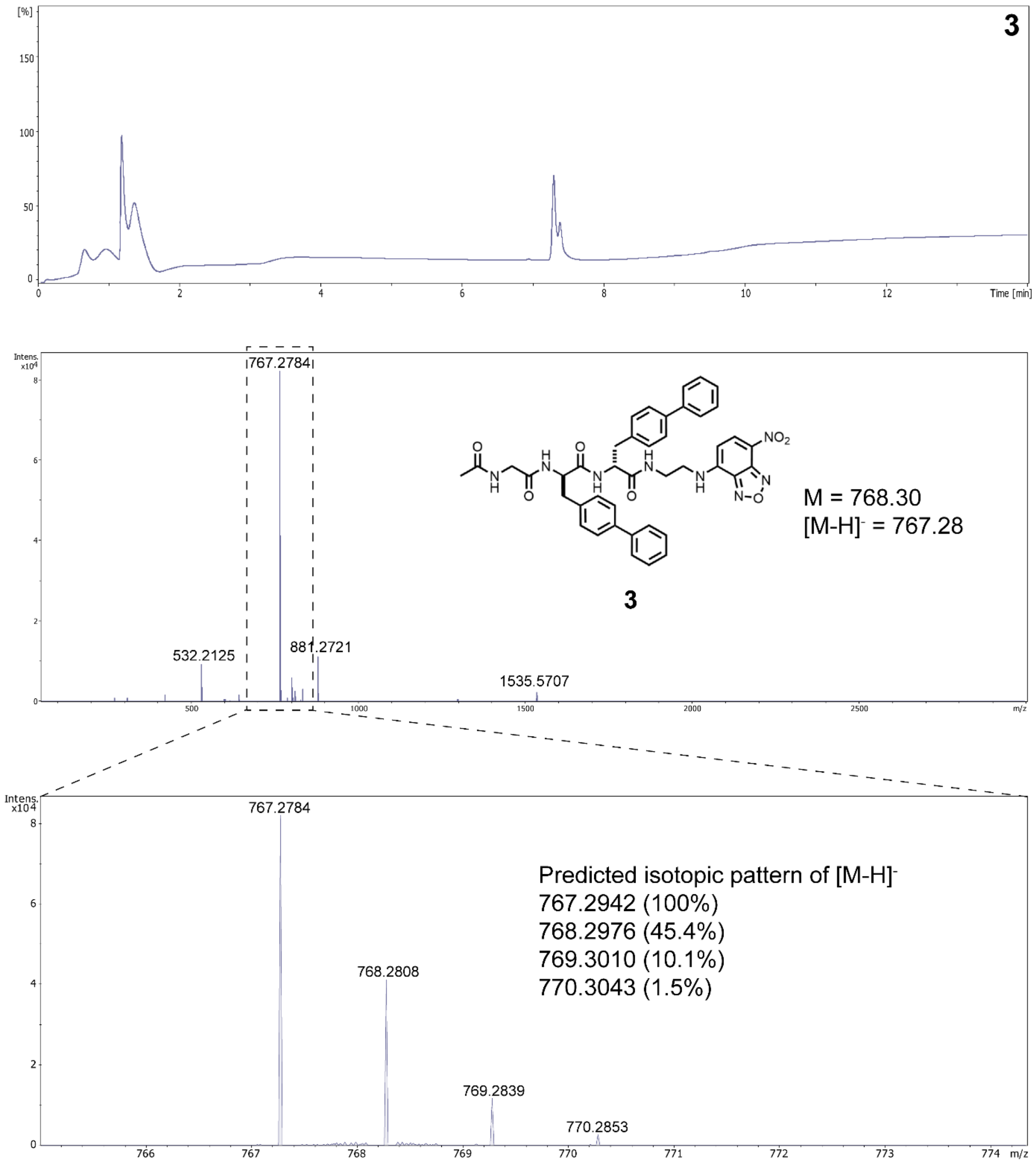

Figure S4. High resolution mass spectrum of AcGbb-NBD (**3**).

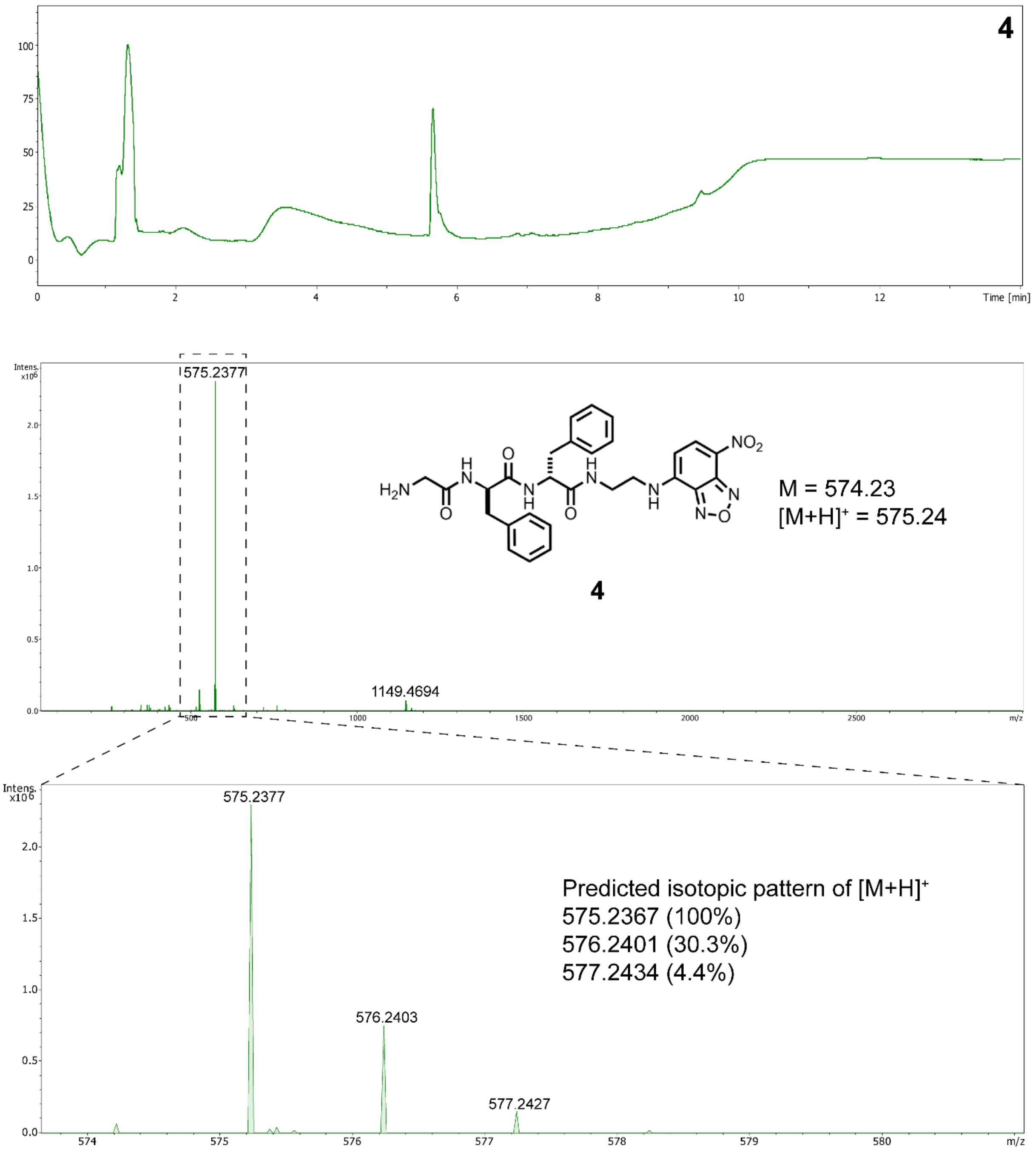

Figure S5. High resolution mass spectrum of Gff-NBD (**4**).

S3. Critical aggregation concentration (CAC) determination

The CAC values were measured using pyrene as a fluorescent probe. Specifically, compounds of different concentrations were prepared in saturated pyrene solution in phenol red-free MEM medium. Fluorescence spectra were recorded on a Biotek Synergy multi-mode microplate reader. The intensity ratio at 378 nm and 393 nm (I_1/I_3) was plotted against compound concentration, and the CAC value was obtained from the concentration at the inflection point.

S4. TEM sample preparation

Carbon-coated 400 mesh copper grids were glow discharged, and 5 μL of sample solution was applied to each grid. After 30 s, the solution was blotted off, and the grids were rinsed twice with ddH_2O followed by a single rinse with uranyl acetate. The grids were then stained with uranyl acetate, excess stain was removed with filter paper, and the samples were air-dried before imaging.

S5. Cell culture

HeLa and SH-SY5Y cell lines were obtained from the American Type Culture Collection (ATCC, Manassas, VA, USA). HeLa cells were maintained in MEM supplemented with 10% FBS and 1% penicillin–streptomycin (100 U/mL penicillin, 100 μg/mL streptomycin). SH-SY5Y cells were cultured in a 1:1 mixture of EMEM and F-12 medium containing 10% FBS and 1% penicillin–streptomycin.

S6. MTT assays

Cells were seeded into 96-well plates at a density of 1 × 10^4 cells per well and cultured for 24 h. The medium was then replaced with fresh medium containing varying concentrations of compounds. After 24, 48, or 72 h of treatment, the compound-containing medium was removed, and 10 μL of MTT solution (5 mg/mL, ACROS Organics) was added to each well for an additional 4 h incubation at 37 °C. Subsequently, 100 μL of SDS-HCl solution was added to terminate the reaction and solubilize the formazan. Absorbance at 595 nm was measured using a DTX880 Multimode Detector. Data were collected in triplicate (n = 3), and results were expressed as the percentage of cell viability relative to untreated controls.

S7. Confocal laser scanning microscopy (CLSM) imaging

Confocal dishes (35 mm with a 20 mm #1.5 glass bottom well, Cellvis) were used to prepare samples for CLSM. For live-cell imaging, exponentially growing cells were seeded at a density of 1.0 × 10^5 cells per dish and cultured for 24 h. The culture medium was then replaced with fresh medium containing the compound of interest, and the cells were incubated at 37 °C in a humidified 5% CO_2 atmosphere for the indicated period. Samples were then imaged using a Nikon AX-R CLSM.

For time-lapse imaging, exponentially growing cells were seeded at the same density on confocal dishes and incubated for 24 h. The samples were washed three times with Live Cell Imaging Solution, stained with Hoechst 33342 for 10 min, and washed again four times to eliminate excess dye. Imaging was performed on a Nikon AX-R CLSM, with cell positions and the laser focal plane determined by nuclear fluorescence using the 405 nm laser. The Nikon

Perfect Focus System was engaged to prevent focus drift. Afterward, the imaging solution was replaced with fresh imaging solution containing the compound of interest, and CLSM images from multiple channels were acquired immediately. Time-series imaging was performed at 1-min intervals for 40 cycles, and the resulting fluorescence images were saved for further analysis.

S8. Cell click reaction

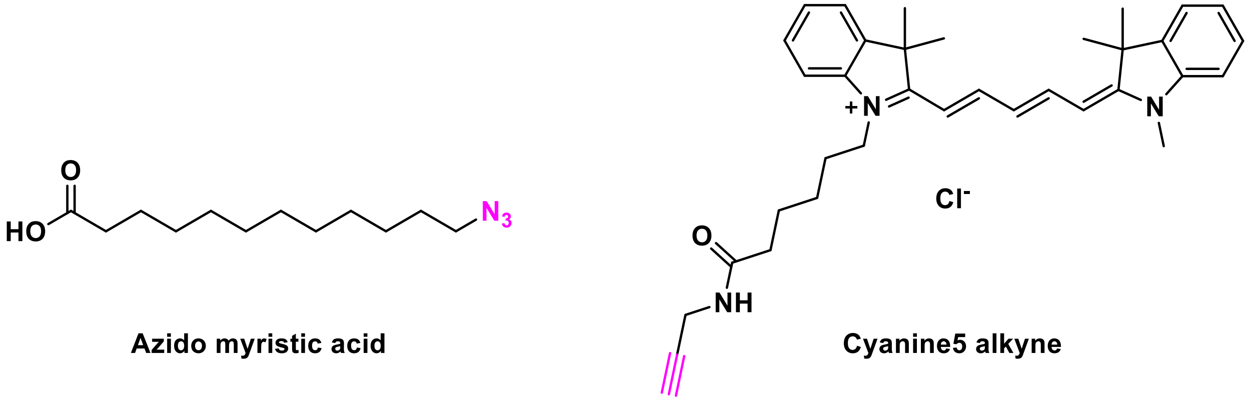

Scheme S3. Chemical structures of azido myristic acid and Cyanine5 alkyne.

Cells in the exponential growth phase were seeded into 96-well glass-bottom confocal plates at a density of 1.0 × 10^4 cells per well and cultured for 24 h. Following removal of the medium, fresh medium supplemented with 50 μM azido myristic acid was added, and the cells were incubated for another 24 h at 37 °C in a humidified 5% CO_2 atmosphere. The cells were then fixed with 4% paraformaldehyde in PBS for 10 min, followed by three washes with PBS. Permeabilization was carried out using PBS containing 0.25% Triton X-100 for 10 min, after which the cells were washed three times with PBS and once with 2% BSA in PBS. The click

reaction mixture, prepared from reaction buffer, copper (II) sulfate, reducing agent, and Cy5 alkyne according to the manufacturer's instructions (Click-&-Go® Cell Reaction Buffer Kit, Vector Laboratories), was applied to the cells for 30 min at room temperature in the dark. Finally, the cells were washed once with 2% BSA in PBS and examined using a Nikon AX-R CLSM.

S9. Plasmid transfection

96 well glass bottom plates (#1.5 cover glass, Cellvis) were used to prepare samples for plasmid transfection. Cells were seeded at a density of 8×10^3 per well and cultured for 24 h to allow attachment. When they reached 40–50% confluency, transfection was carried out using the Xfect™ Transfection Reagent. Briefly, 3 μg of plasmid DNA was mixed with Xfect Reaction Buffer, followed by the addition of 1 μL Xfect Polymer. The mixture was gently vortexed and incubated at room temperature for 10 min to form nanoparticles. 10 μL of the complex solution was then added dropwise to the culture medium in each well, and the plate was gently rocked. Cells were incubated overnight at 37 °C, after which the medium was replaced with fresh culture medium and incubation continued for an additional 48 h. The cells were then prepared for live-cell imaging.

S10. Detection of peptide myristoylation by HR-MS

A total of 1.2×10^7 cells were treated with 1 μM of compound **1** or vehicle control (DMSO) for 24 h, then washed twice with HEPES buffer. Cells were collected and centrifuged to obtain

a pellet, which was resuspended in 200 μL of 1× PBS. To this, 1.5 mL of HPLC-grade methanol was added, and the mixture was vortexed for 1 min. Subsequently, 5 mL of methyl tert-butyl ether (MTBE) was added, and the sample was gently rocked for 1 h at room temperature. Following this, 1.2 mL of water was added, vortexed for 1 min, and centrifuged. The upper MTBE phase was collected, while the lower aqueous phase was re-extracted twice with two volumes of MTBE/methanol/water (10:3:2.5, v/v/v). The combined MTBE phases were dried under a nitrogen stream. The remaining residue was dissolved in 150 μL of methanol and submitted for HR-MS analysis.

S11. Drug resistance test

HeLa cells were split into two groups for culture. In Group 1, cells were treated with compound **1** (500 nM) for 24 h, followed by replacement with fresh medium for an additional 2 days to allow surviving cells to recover and proliferate. These cells were then subcultured and grown to 80–90% confluency before being re-exposed to gradually increasing concentrations of **1** (up to 2 μM), undergoing a total of seven additional stimulation cycles. In Group 2, cells were treated with vehicle (DMSO) instead of **1** and subjected to the same procedure as controls. After eight cycles, the surviving stimulated cells from Group 1 and the unstimulated control cells from Group 2 were collected and seeded into 96-well plates at 1×10^5 cells per well for 24 h. The cells were then exposed to compound **1** for 24 h, and viability was assessed using the MTT assay.

S12. Untargeted lipidomics profiling

Lipidomics samples were prepared following a previously reported protocol.[1] 1.2×10^7 HeLa cells were treated with or without **1** (0.5 μM and 5 μM) for 24h. After treatment, cells were harvested at room temperature using trypsin and centrifuged at 1000 g for 5 min. The supernatant was removed, and the pellet was resuspended in 200 μL of 1× PBS. For extraction of non-polar lipids, 1.5 mL of HPLC-grade methanol was added to the pellet and vortexed for 1 min, followed by addition of 5 mL of MTBE and rocking for 1 h at room temperature. Subsequently, 1.2 mL of water was added, vortexed for 1 min, and centrifuged at 1000 g for 10 min. The upper MTBE phase was collected, and the lower aqueous phase was re-extracted with two volumes of MTBE/methanol/water (10:3:2.5, v/v/v). The MTBE fractions were combined, dried under a nitrogen stream, and the resulting lipid extracts were submitted for MS analysis at the BIDMC-Harvard Mass Spectrometry Facility. Data were median-normalized across each sample.

S13. Supplemental figures

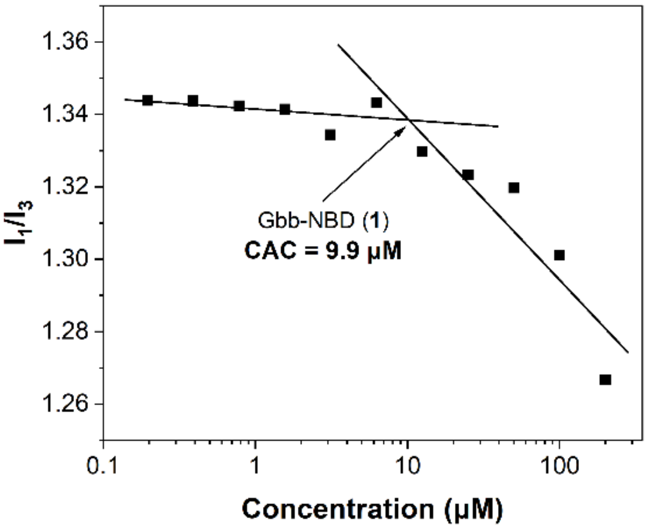

Figure S6. Critical aggregation concentration (CAC) of Gbb-NBD (**1**) determined using pyrene.

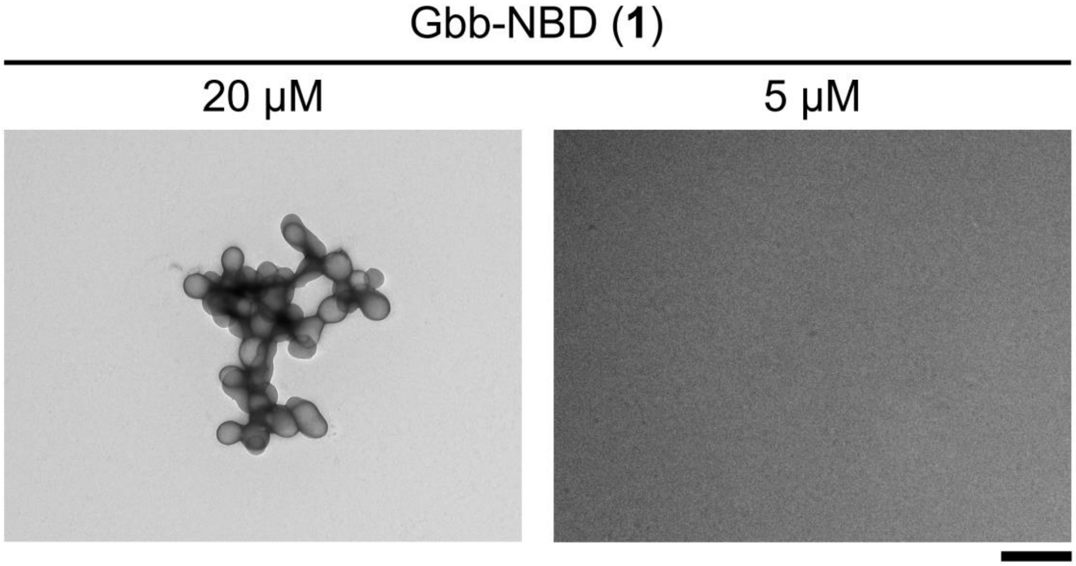

Figure S7. TEM images of 20 and 5 μM of Gbb-NBD (**1**) in ddH_2O at pH 7. (Scale bar = 400 nm)

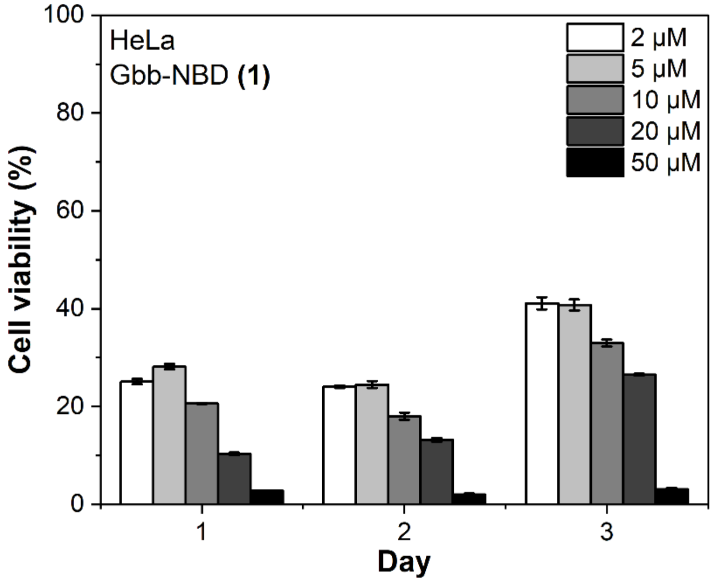

Figure S8. Cell viability of HeLa cells treated with Gbb-NBD (**1**) for 1, 2 and 3 days.

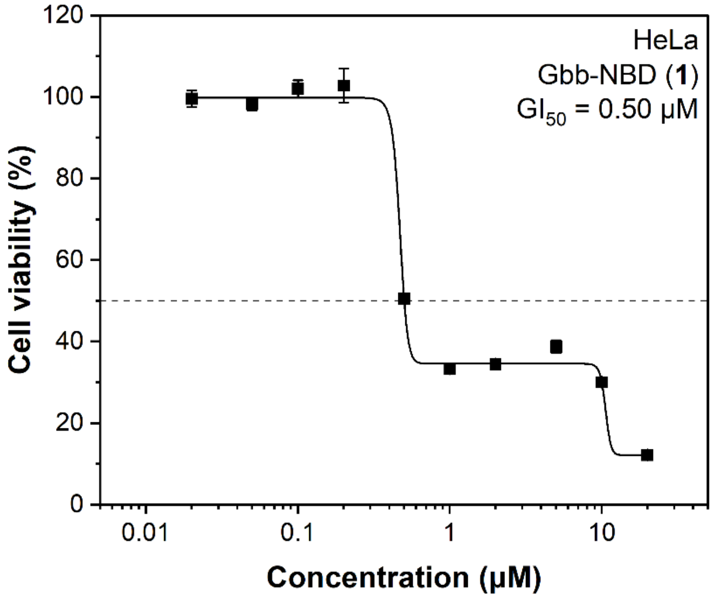

Figure S9. Cell viability of HeLa cells treated with different concentrations of Gbb-NBD (**1**) for 24h to determine its GI_{50}.

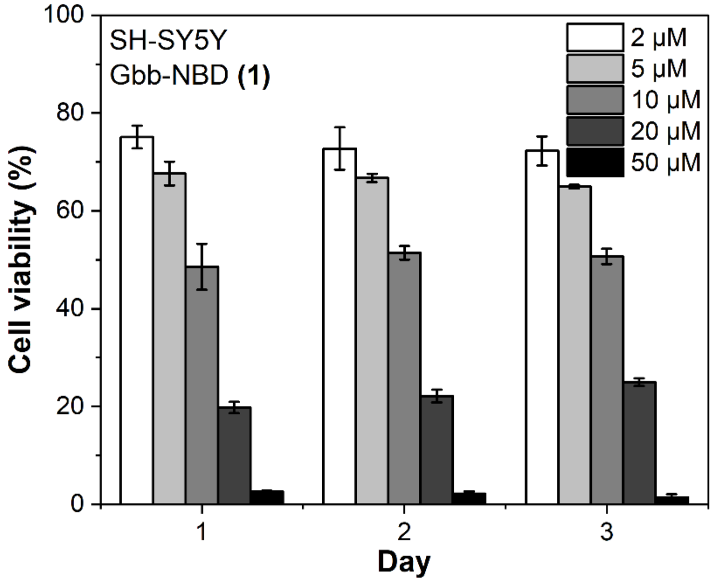

Figure S10 Cell viability of SH-SY5Y cells treated with Gbb-NBD (**1**) for 1, 2 and 3 days.

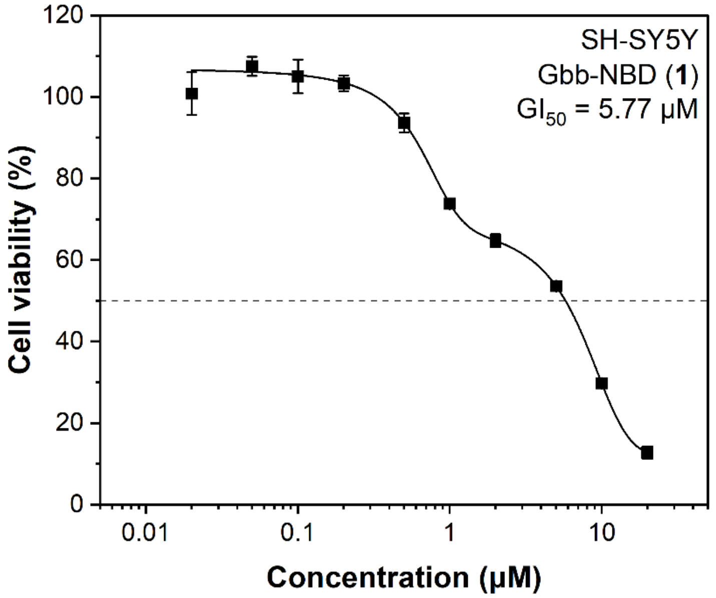

Figure S11. Cell viability of SH-SY5Y cells treated with different concentrations of Gbb-NBD (**1**) for 24h to determine its GI_{50}.

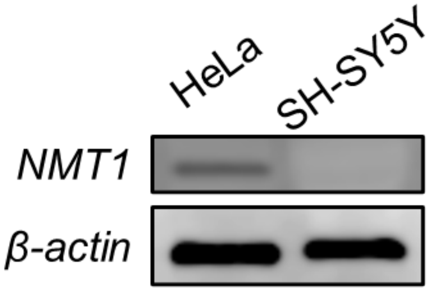

Figure S12. Immunoblotting of NMT1 in HeLa and SH-SY5Y cells.

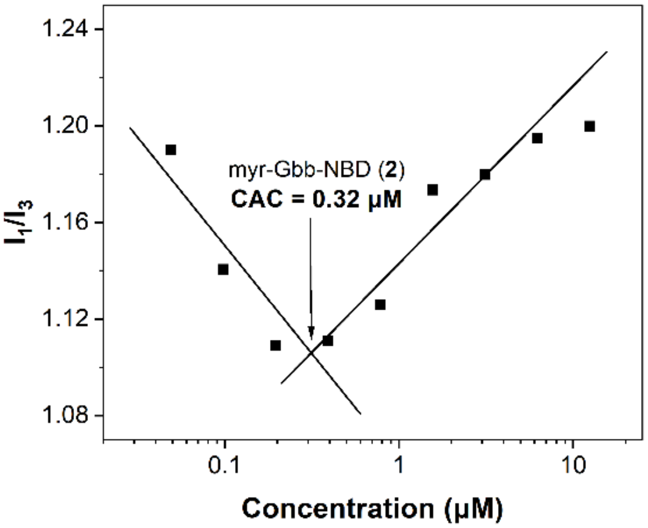

Figure S13. Critical aggregation concentration (CAC) of myr-Gbb-NBD (**2**) determined using pyrene.

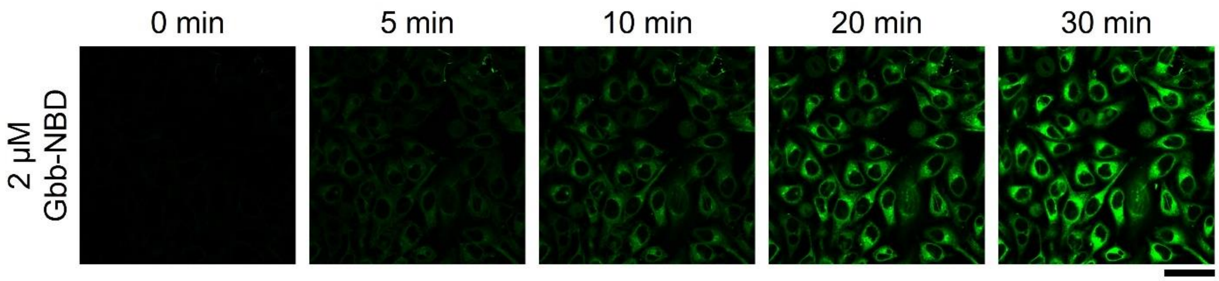

Figure S14. Time-lapse CLSM images of HeLa cells treated with 2 μM Gbb-NBD (**1**). (Scale bar = 50 μm)

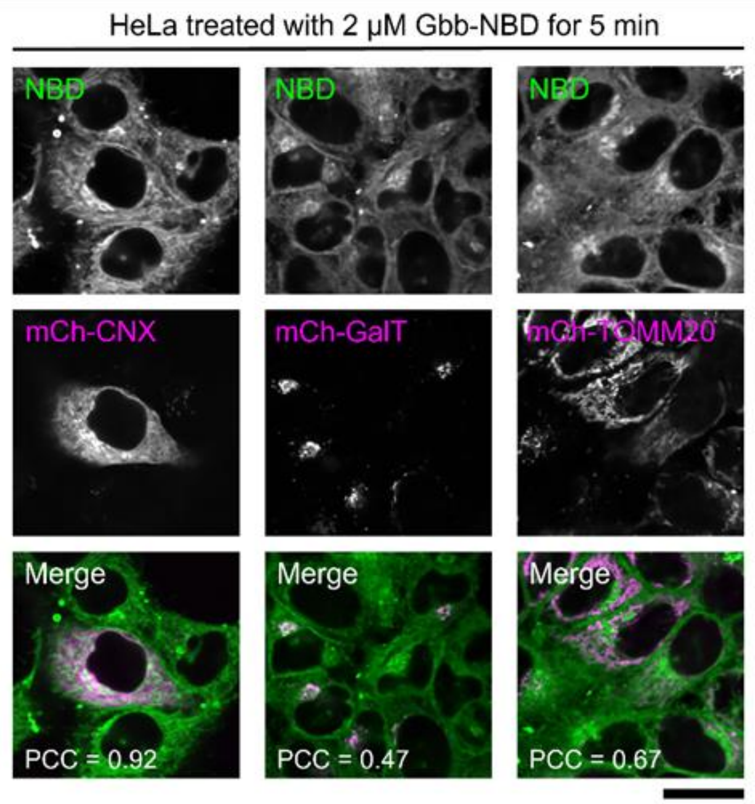

Figure S15. CLSM images of HeLa cells (transfected with mCherry-Calnexin or mCherry-GalT or mCherry-TOMM20) treated with Gbb-NBD (**1**) for 5 min. (Scale bar = 20 μm)

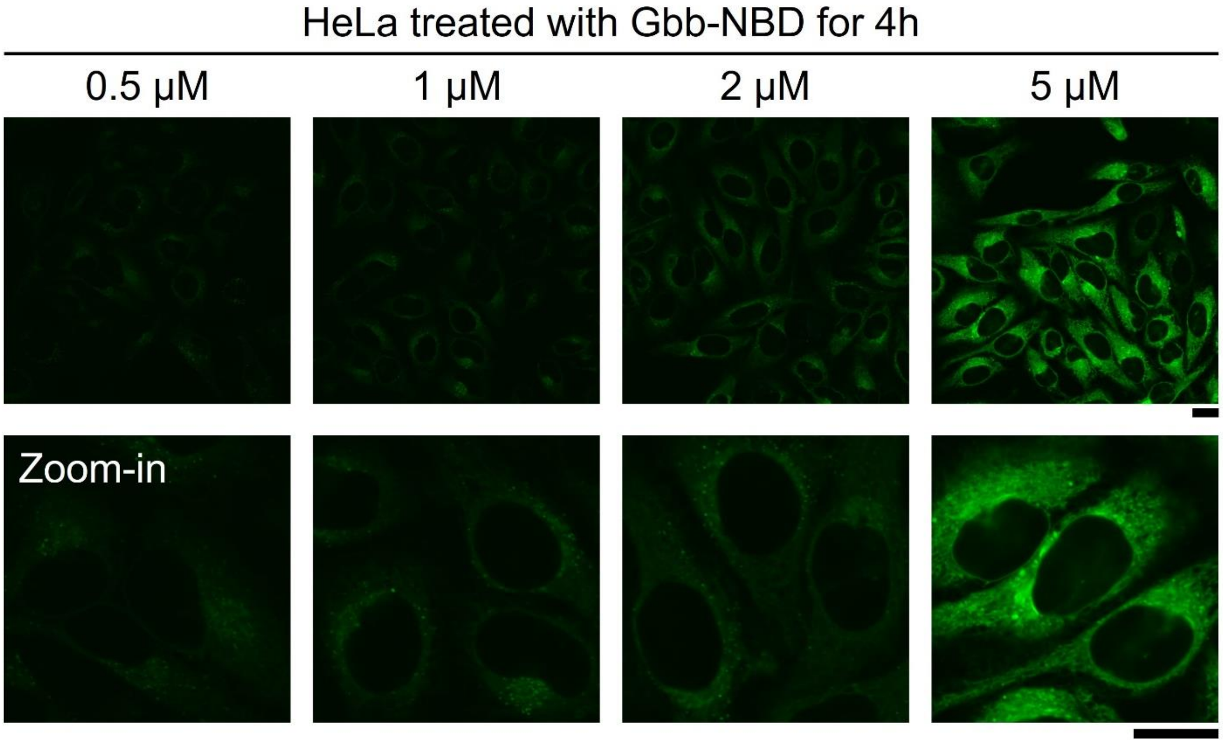

Figure S16. CLSM images of HeLa cells treated with Gbb-NBD (**1**) for 4h. (Scale bar = 20 μm)

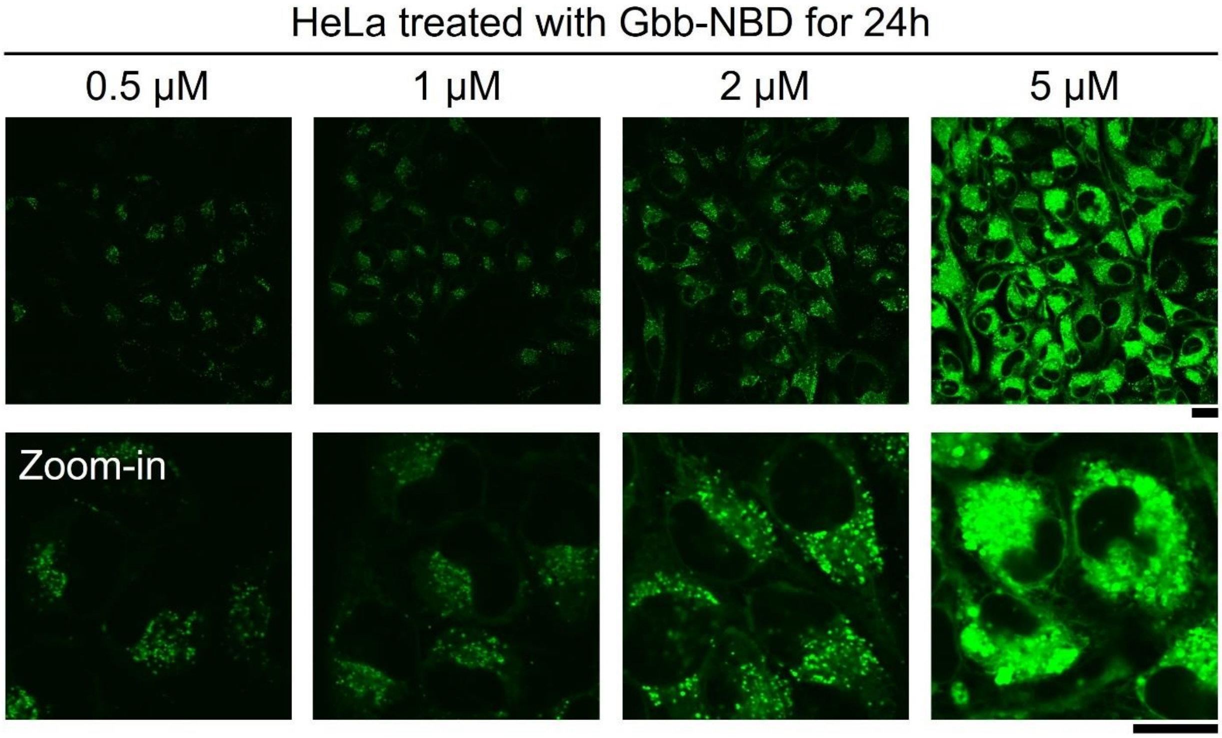

Figure S17. CLSM images of HeLa cells treated with Gbb-NBD (**1**) for 24h. (Scale bar = 20 μm)

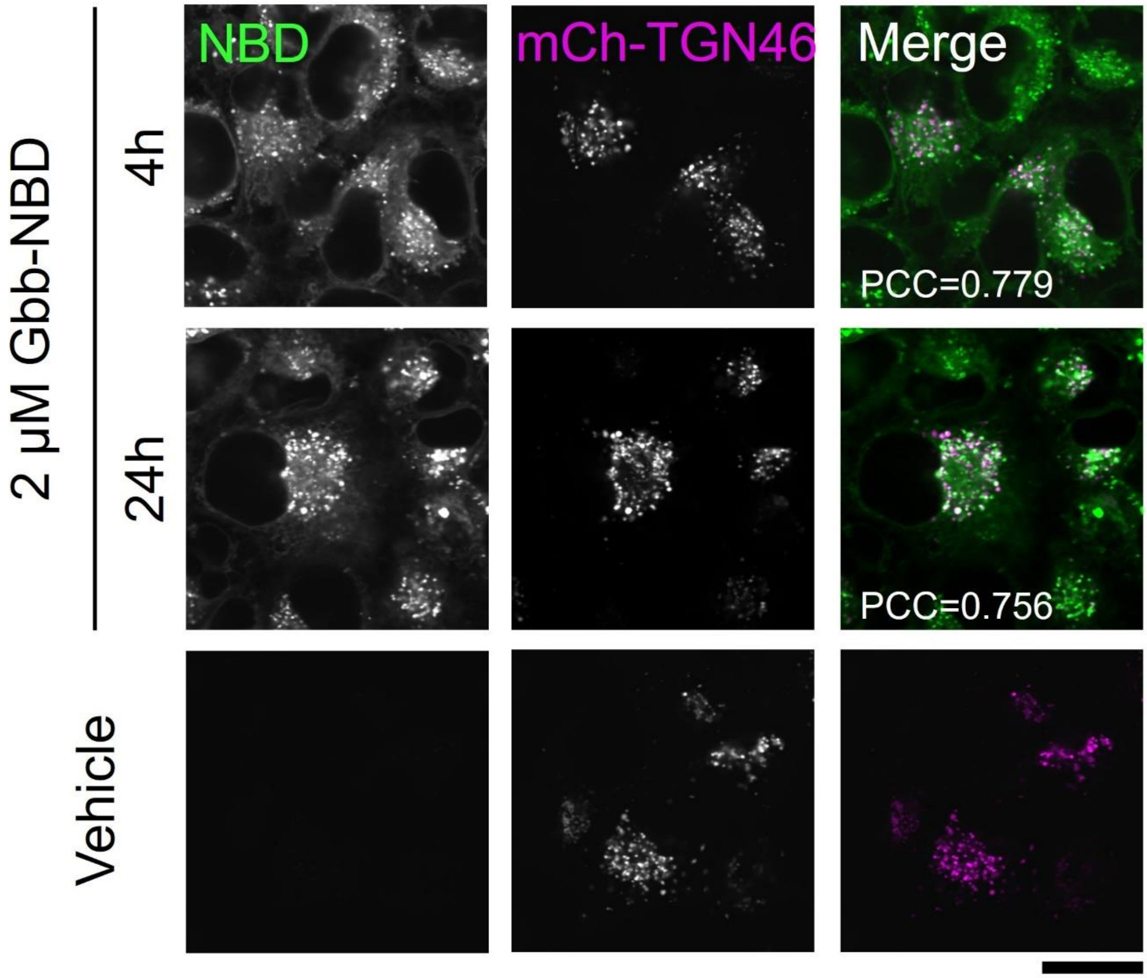

Figure S18. CLSM images of HeLa cells (transfected with mCherry-TGN46) treated with Gbb-NBD (**1**) for 4h and 24h. (Scale bar = 20 μm)

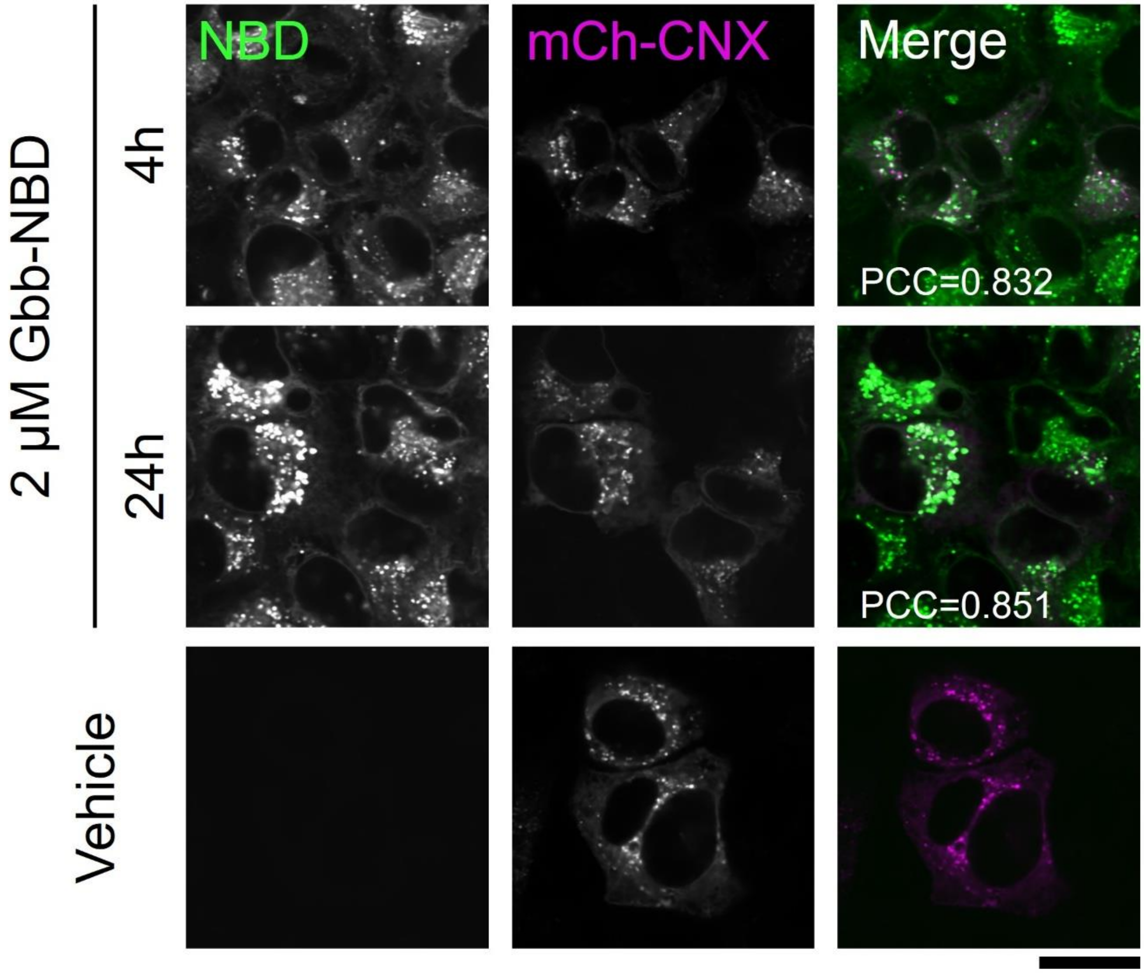

Figure S19. CLSM images of HeLa cells (transfected with mCherry-Calnexin) treated with Gbb-NBD (**1**) for 4h and 24h. (Scale bar = 20 μm)

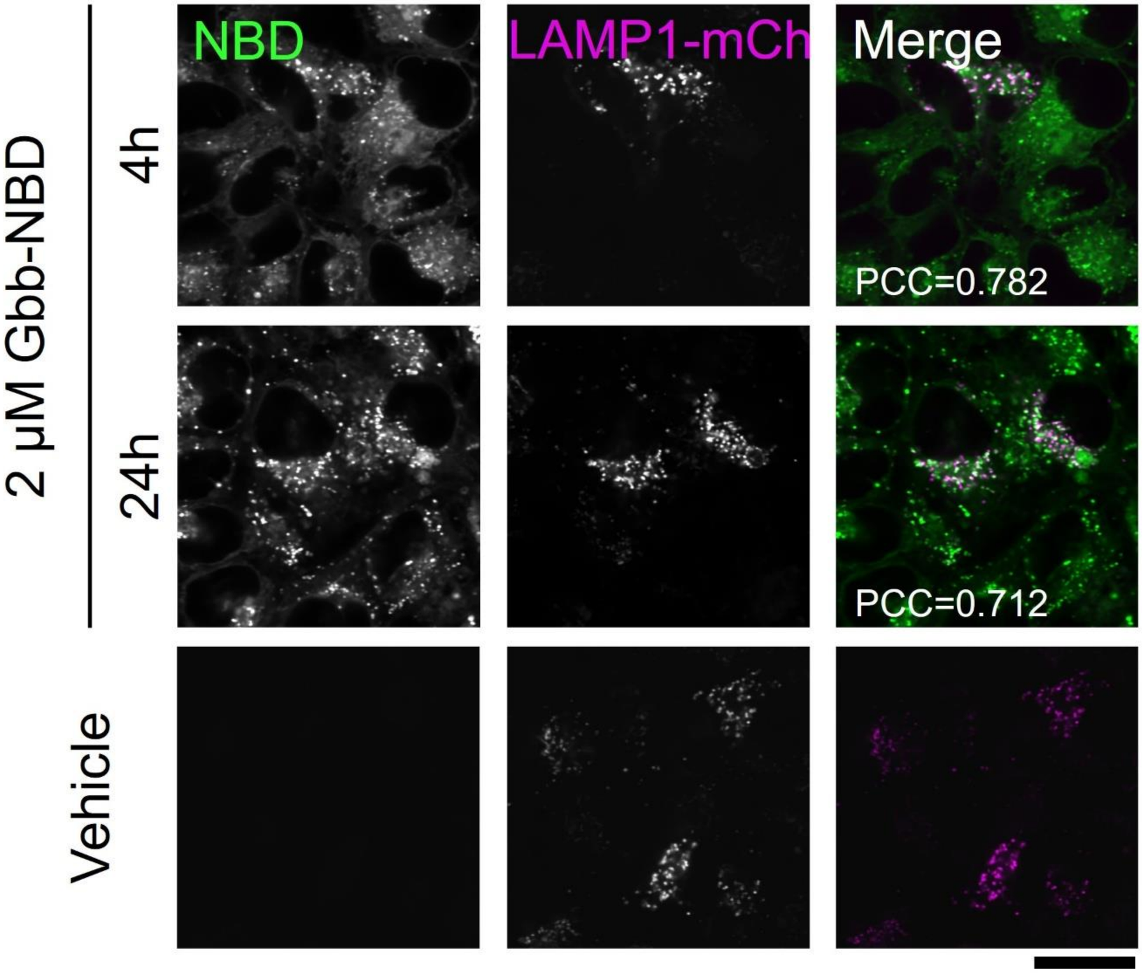

Figure S20. CLSM images of HeLa cells (transfected with LAMP1-mCherry) treated with Gbb-NBD (**1**) for 4h and 24h. (Scale bar = 20 μm)

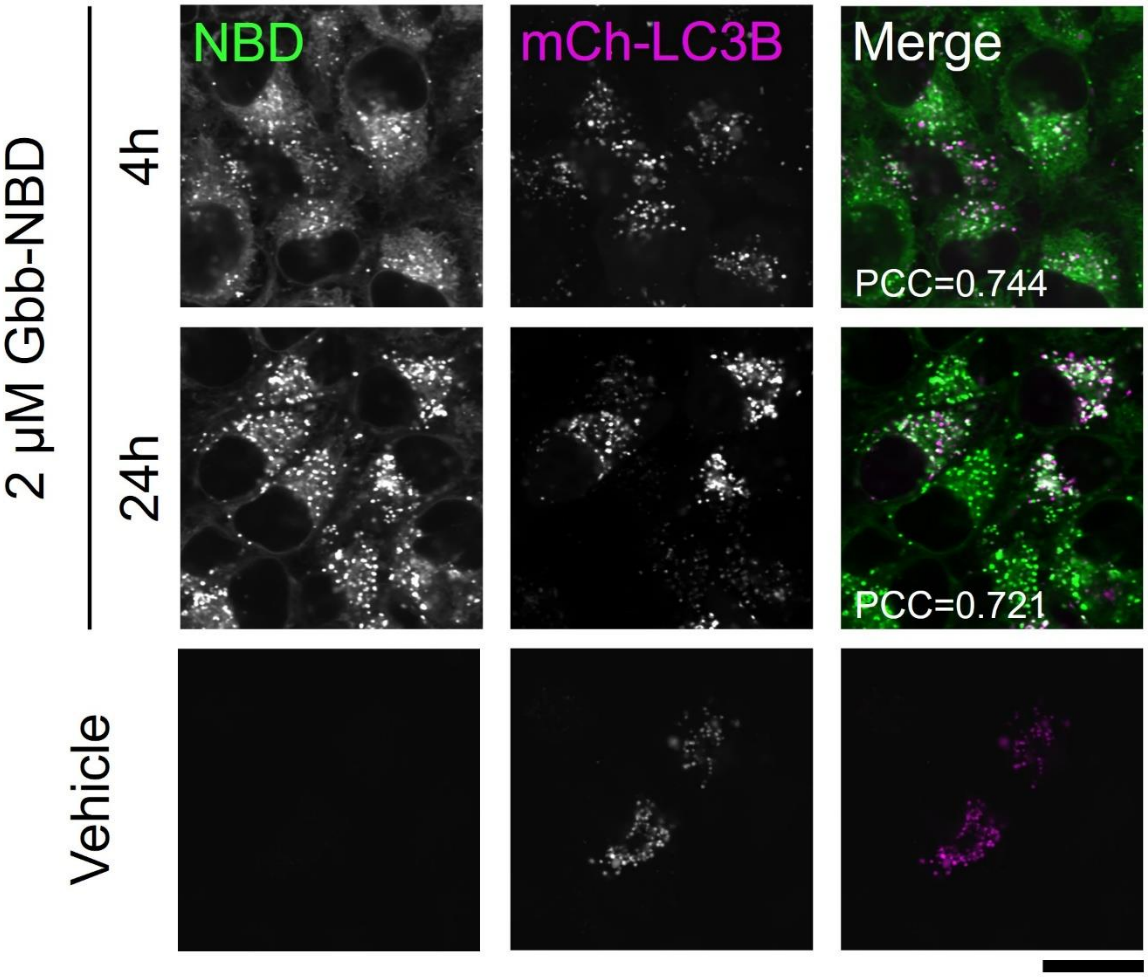

Figure S21. CLSM images of HeLa cells (transfected with mCherry-LC3B) treated with Gbb-NBD (**1**) for 4h and 24h. (Scale bar = 20 μm)

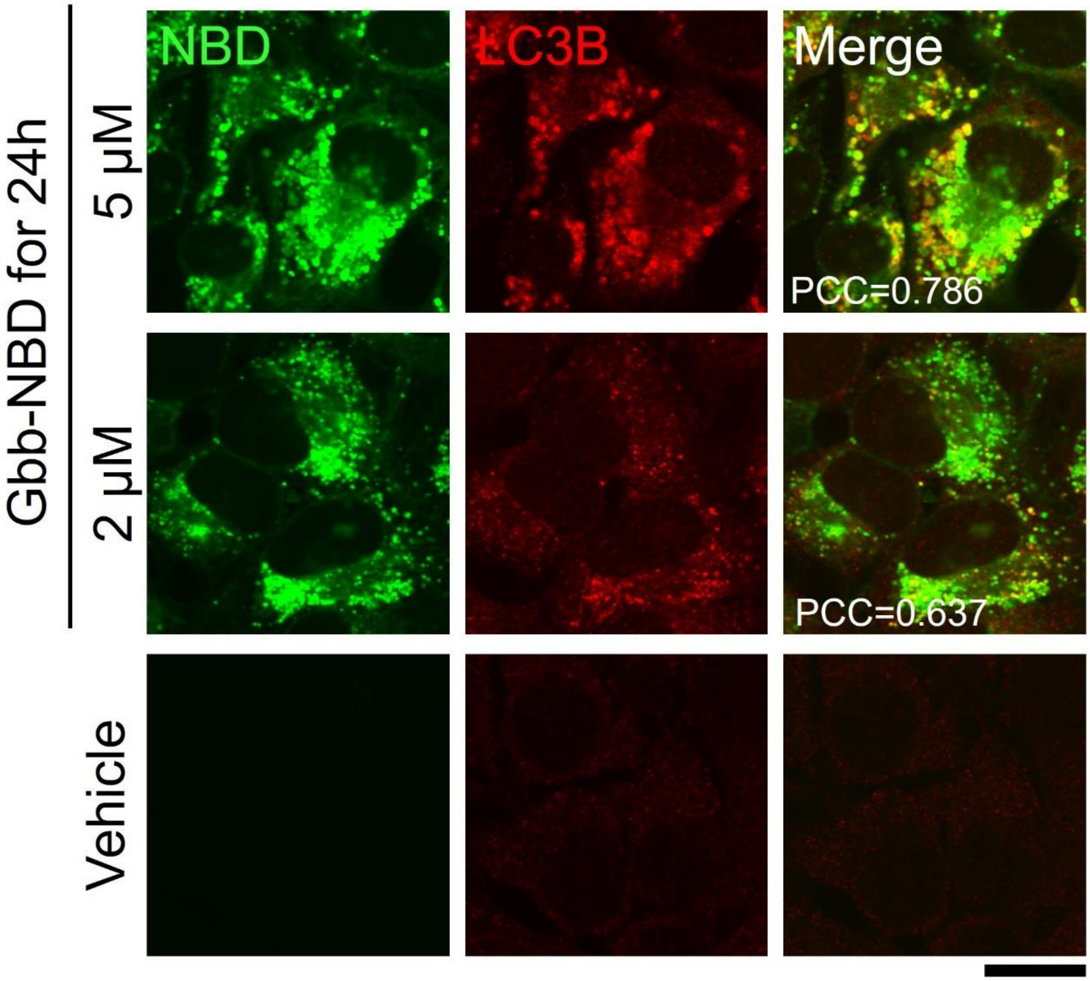

Figure S22. CLSM images of HeLa cells treated with different concentrations of Gbb-NBD (**1**) for 24h and stained for LC3B. (Scale bar = 20 μm)

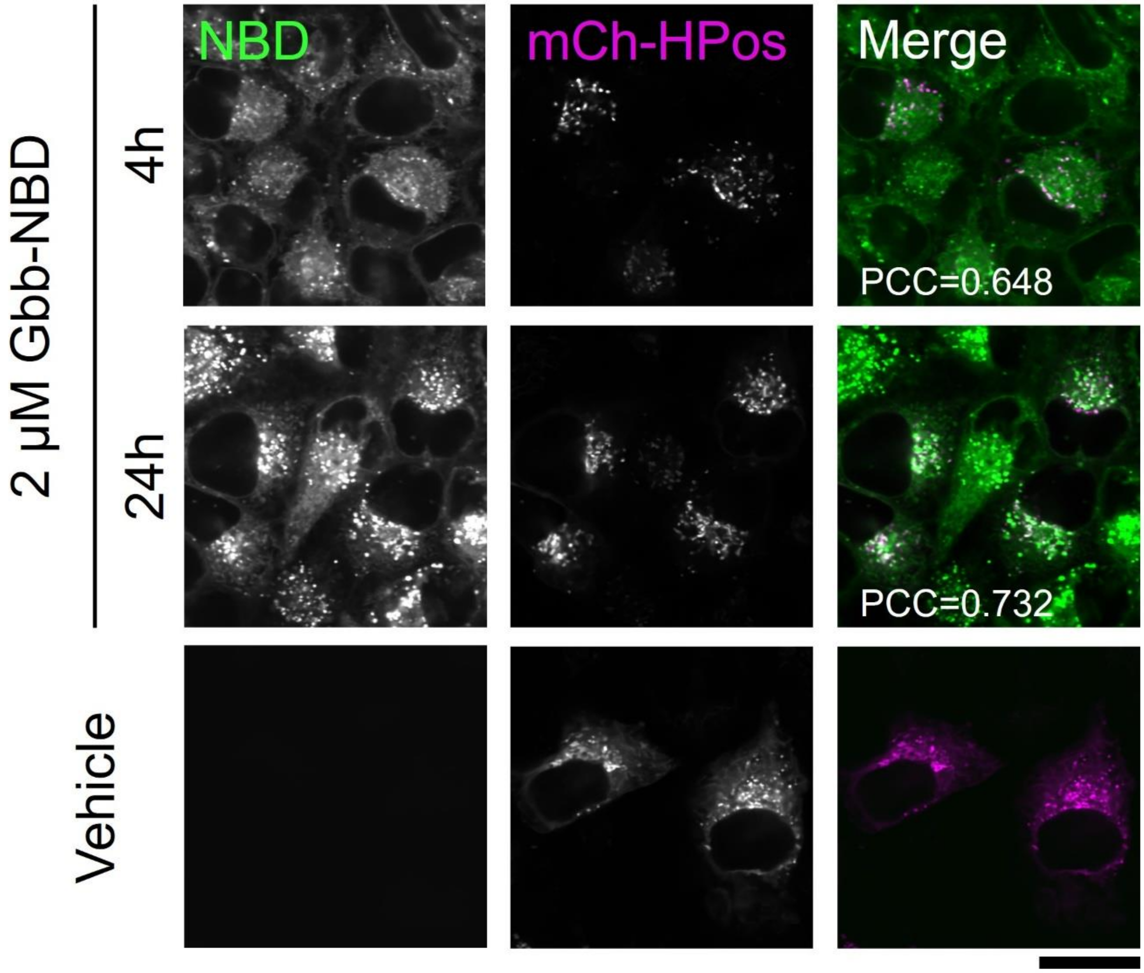

Figure S23. CLSM images of HeLa cells (transfected with mCherry-HPos) treated with Gbb-NBD (**1**) for 4h and 24h. (Scale bar = 20 μm)

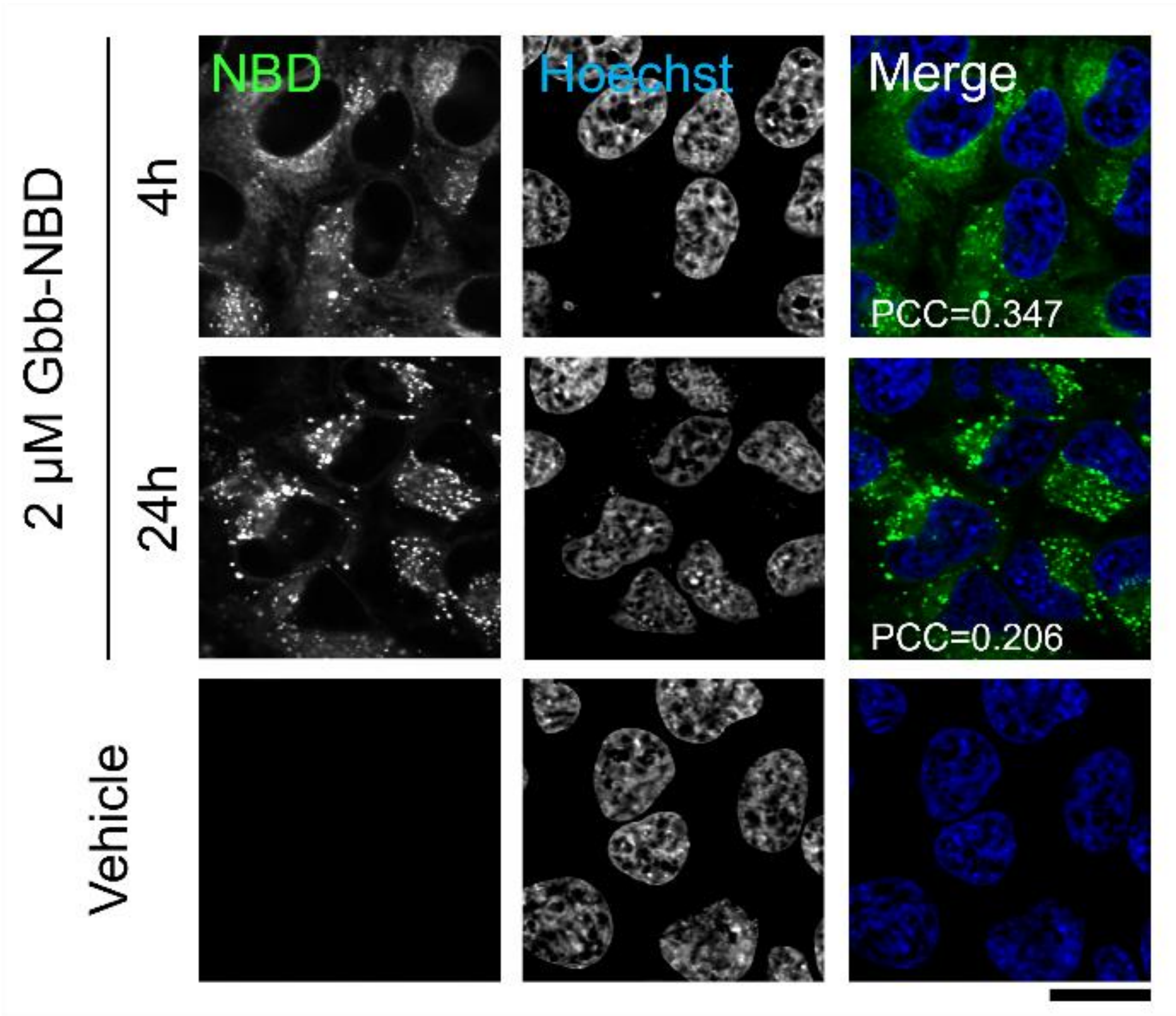

Figure S24. CLSM images of HeLa cells (stained with Hoechst 33342) treated with Gbb-NBD (**1**) for 4h and 24h. (Scale bar = 20 μm)

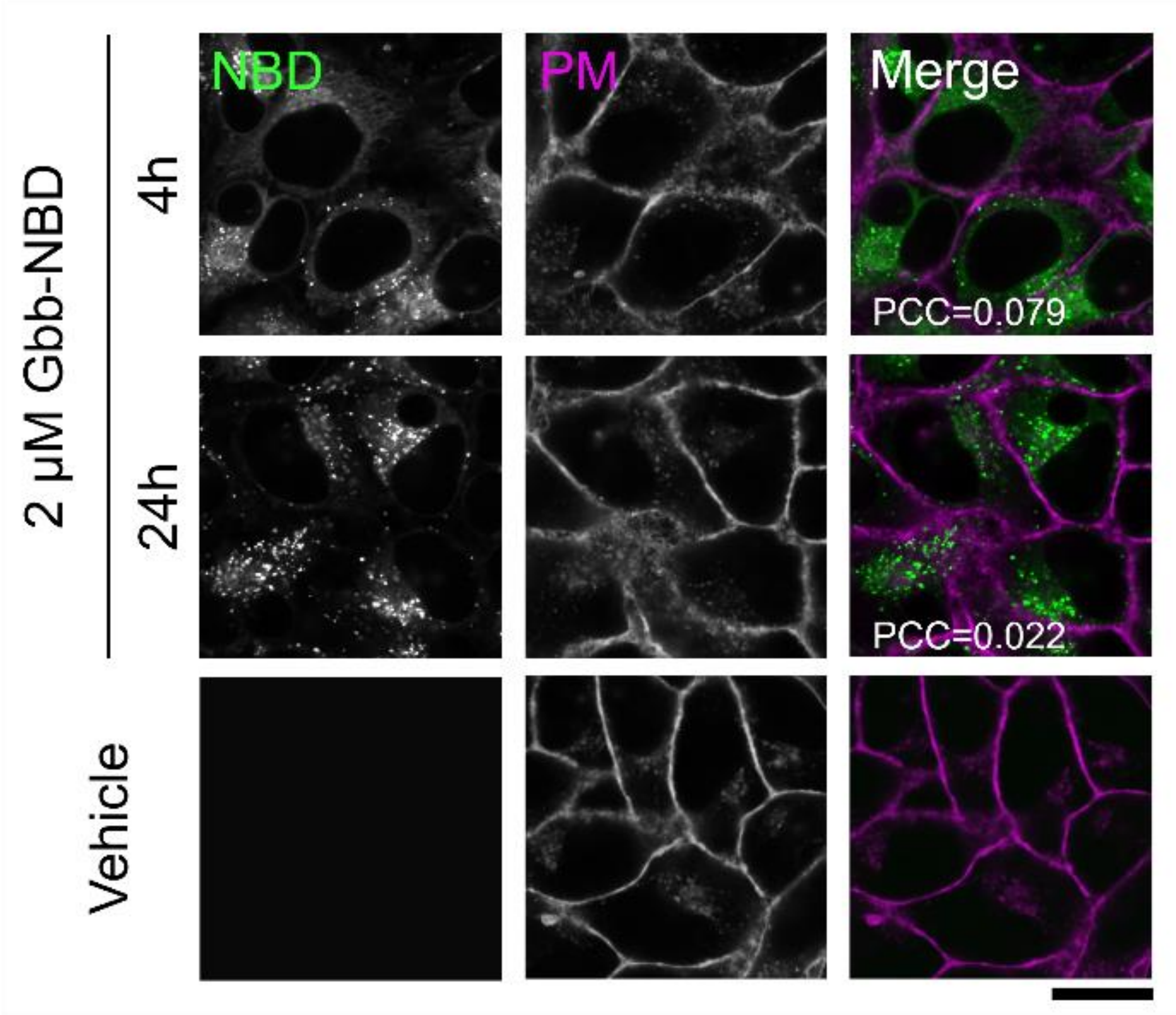

Figure S25. CLSM images of HeLa cells (stained with CellMask™ Plasma Membrane) treated with Gbb-NBD (**1**) for 4h and 24h. (Scale bar = 20 µm)

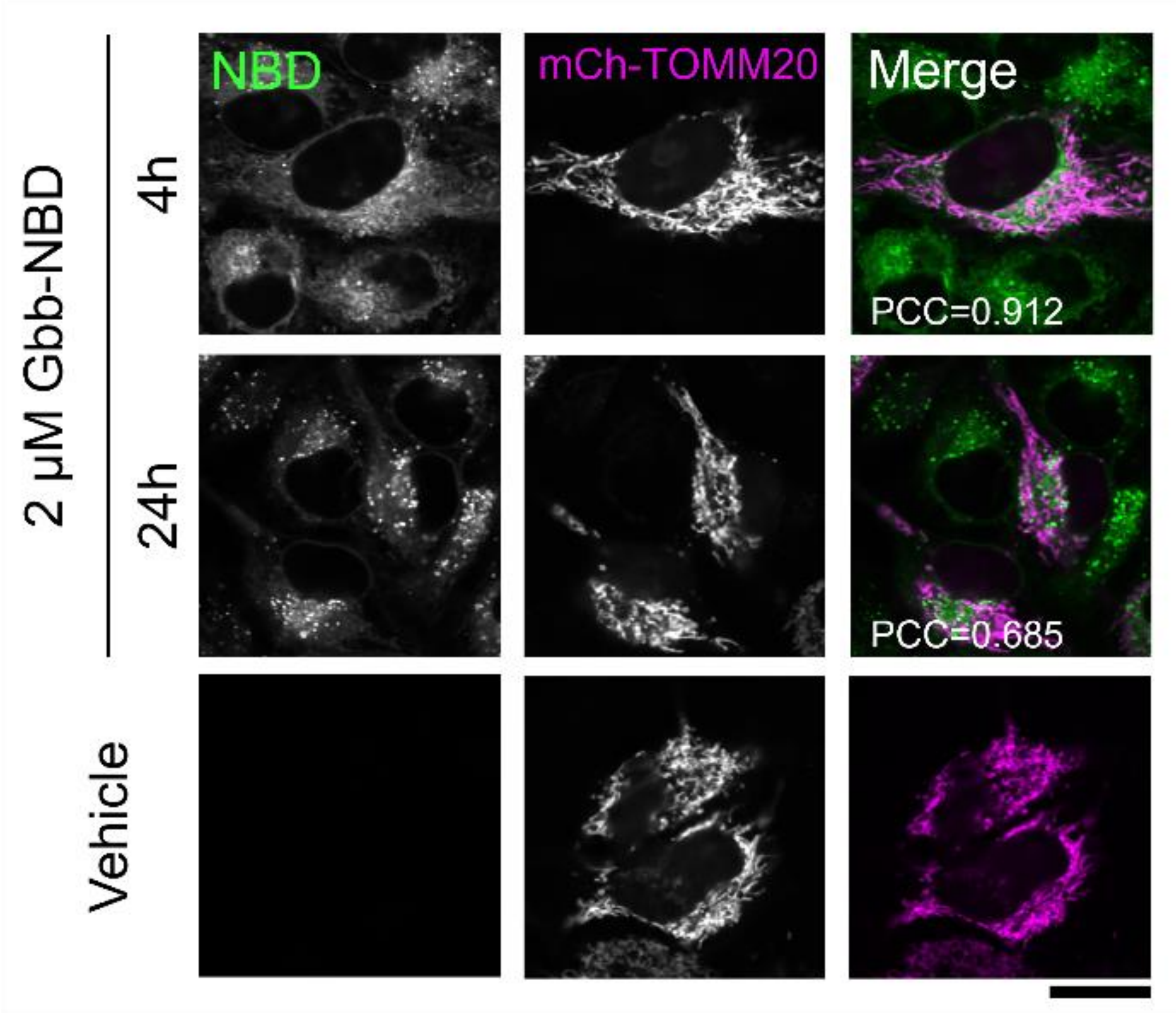

Figure S26. CLSM images of HeLa cells (transfected with mCherry-TOMM20) treated with Gbb-NBD (**1**) for 4h and 24h. (Scale bar = 20 µm)

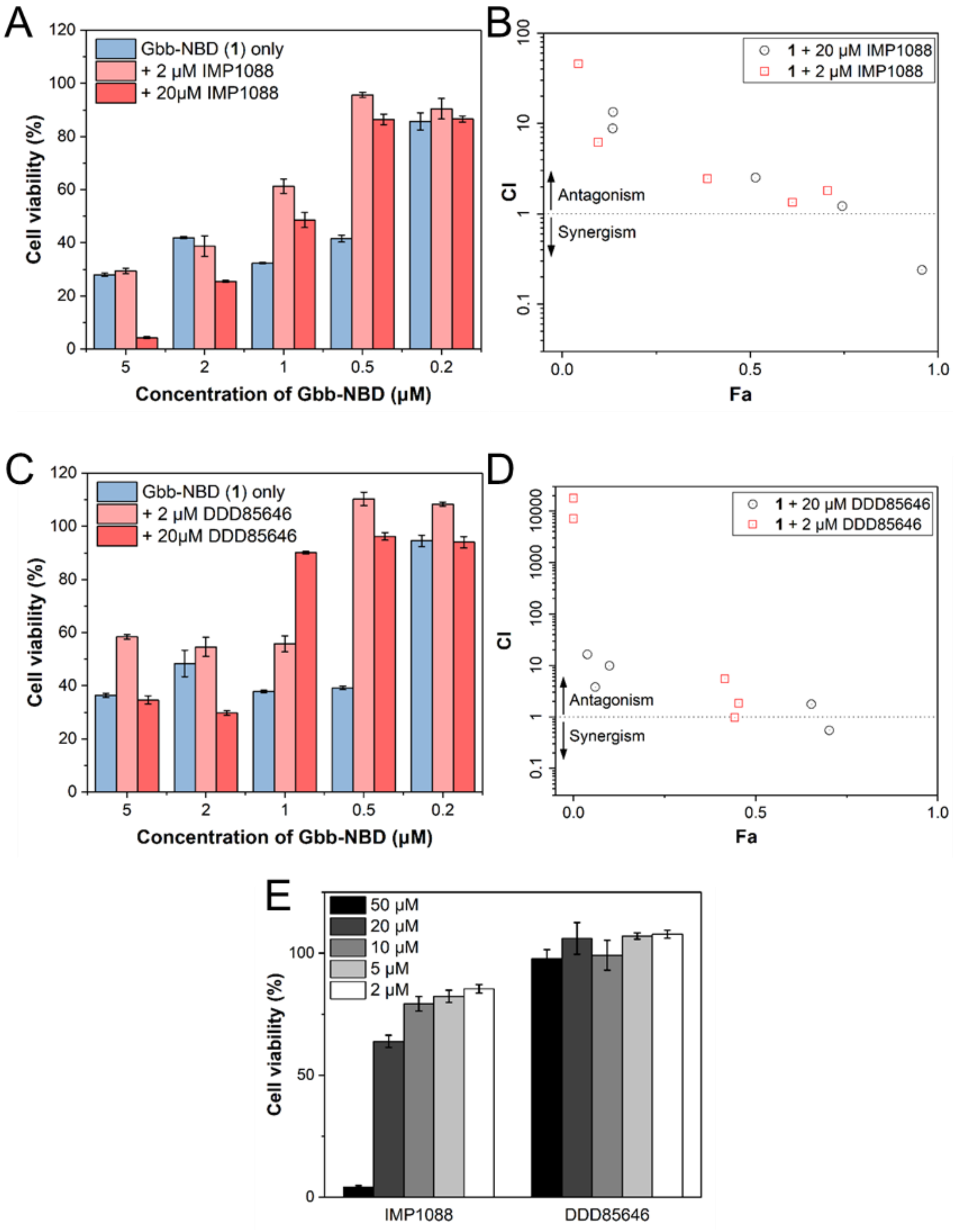

Figure S27. Cell viability of HeLa cells treated with Gbb-NBD with or without the addition of (A) IMP1088 and (C) DDD85646 for 24h, and (B) (D) corresponding combination index (CI) values. (E) Cell viability of HeLa cells treated with IMP1088 or DDD85646 only for 24h.

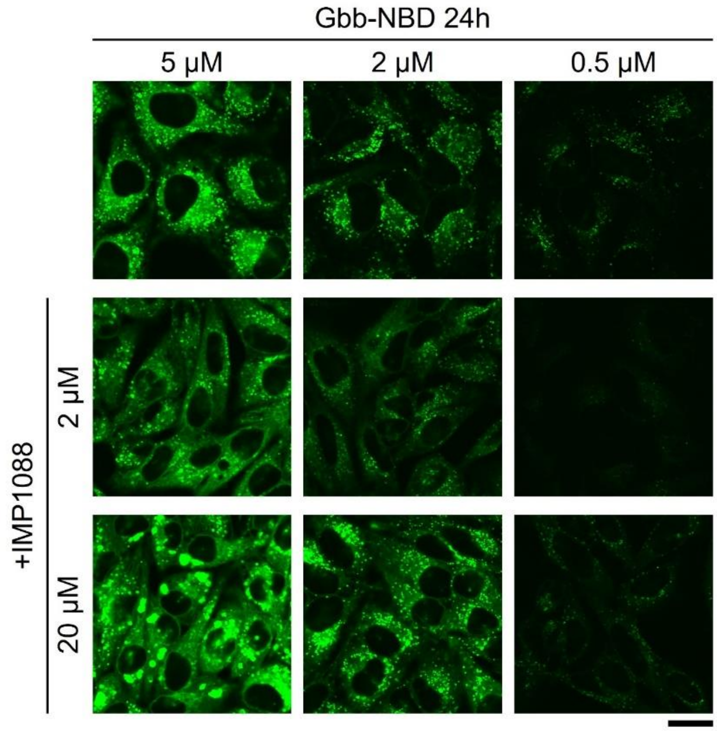

Figure S28. CLSM images of HeLa cells treated with different concentrations of Gbb-NBD (**1**) for 24h with or without IMP1088. (Scale bar = 20 μm)

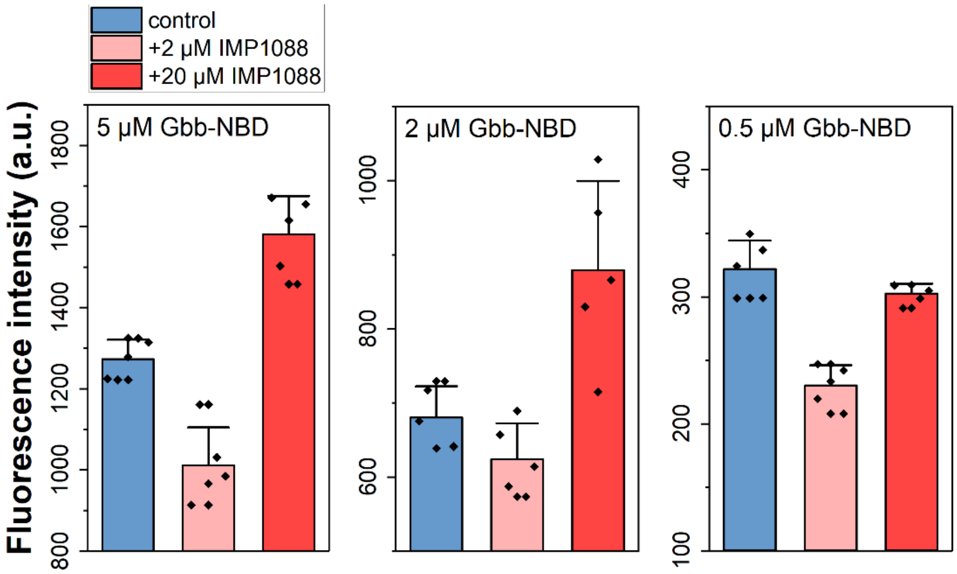

Figure S29. Single-cell quantification of fluorescence intensity in **Figure S28**. (n = 5)

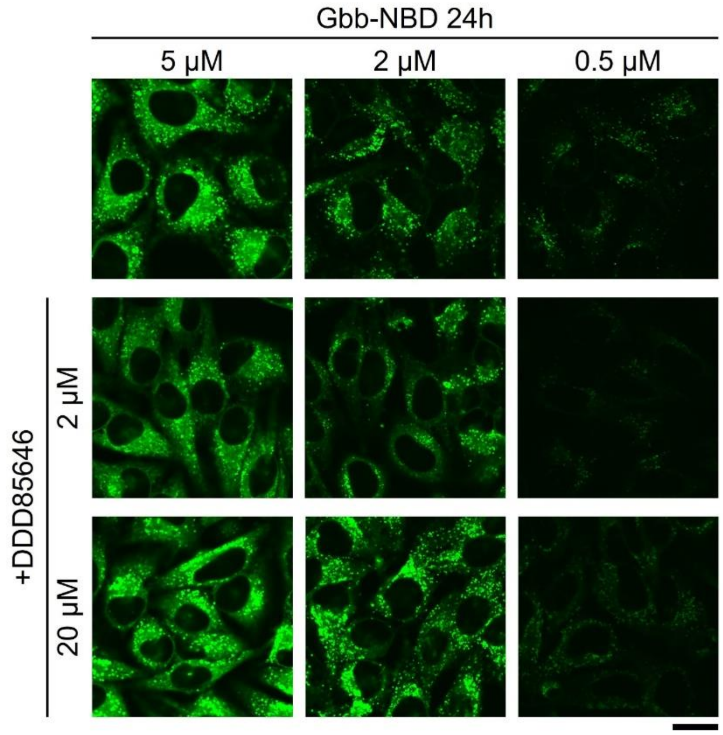

Figure S30. CLSM images of HeLa cells treated with different concentrations of Gbb-NBD (**1**) for 24h with or without DDD85646. (Scale bar = 20 μm)

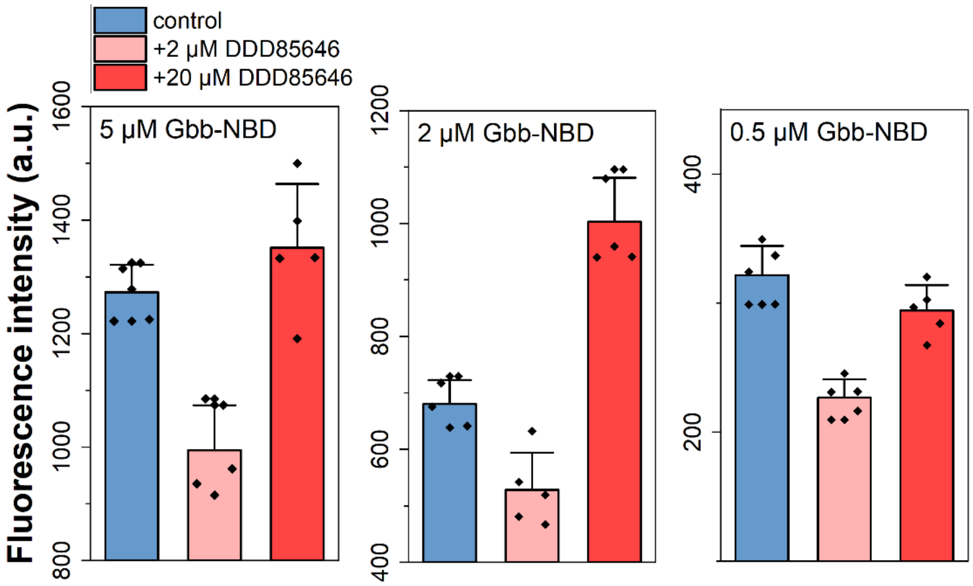

Figure S31. Single-cell quantification of fluorescence intensity in **Figure S30**. (n = 5)

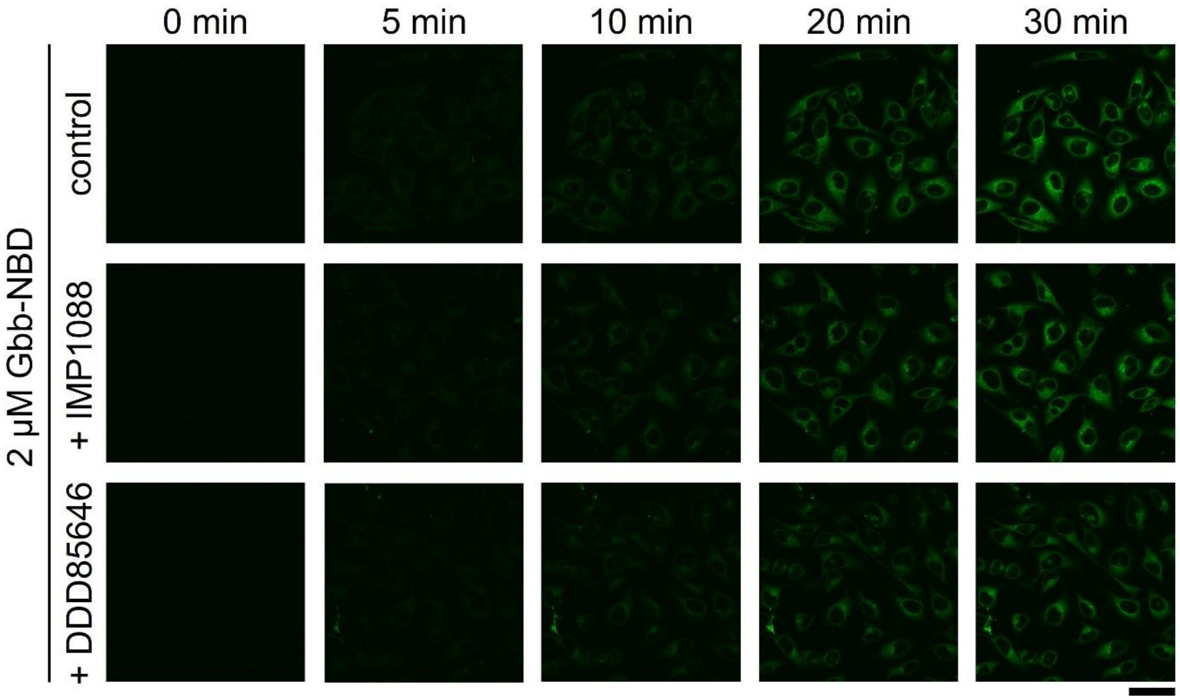

Figure S32. Time-lapse CLSM images of HeLa cells treated with Gbb-NBD (**1**) with or without NMT inhibitors, IMP1088 and DDD85646. (Scale bar = 50 μm)

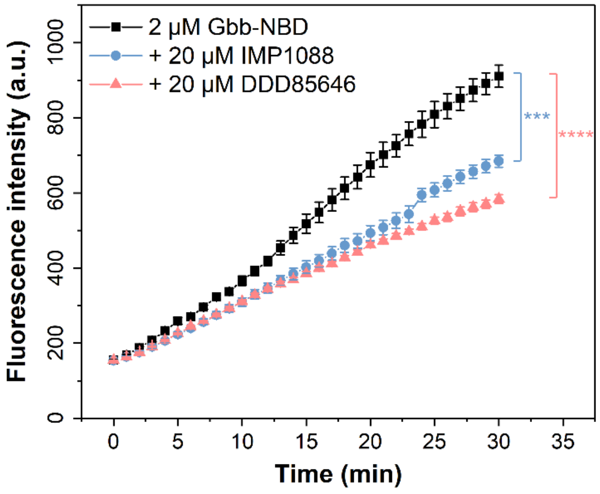

Figure S33. Single-cell fluorescence quantification of fluorescence intensity in **Figure S32**. (n = 5)

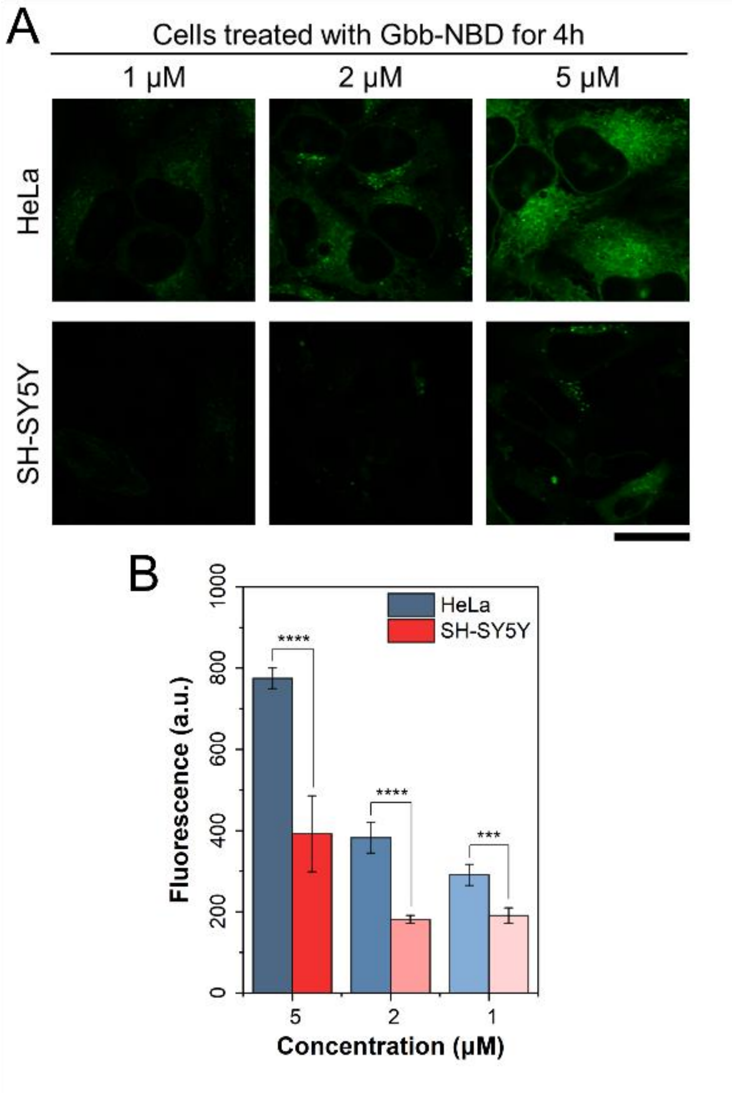

Figure S34. (A) CLSM images of HeLa and SH-SY5Y cells treated with different concentrations of Gbb-NBD (**1**) for 4h and (B) single-cell quantification of fluorescence intensity in (A). (Scale bar = 20 μm)

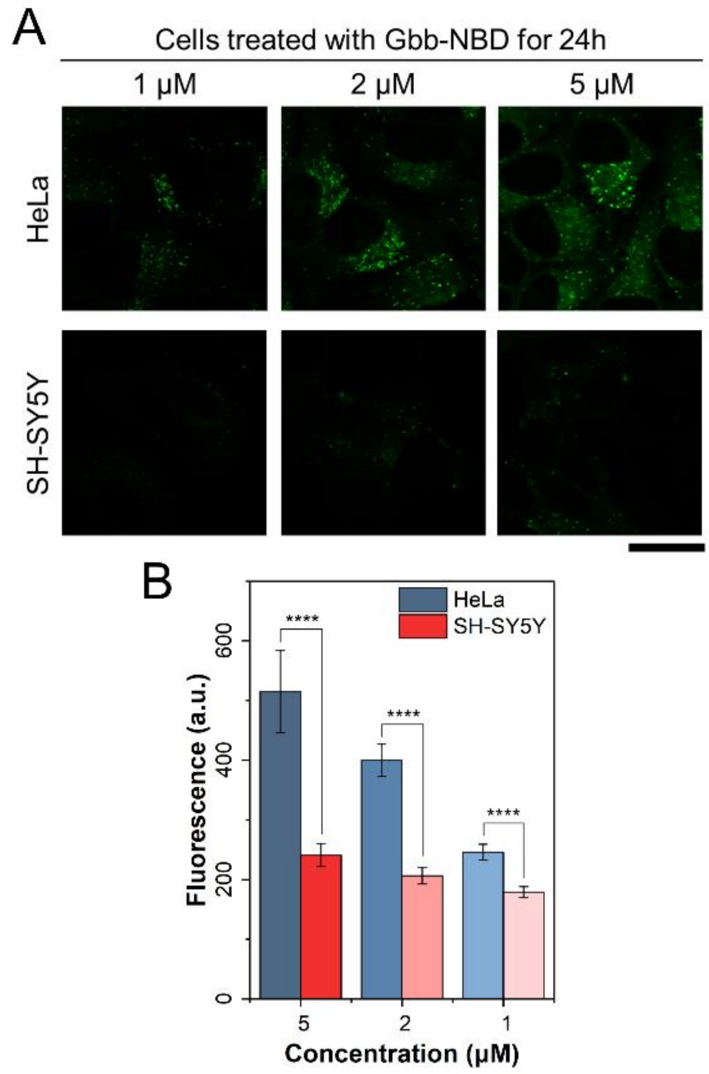

Figure S35. (A) CLSM images of HeLa and SH-SY5Y cells treated with different concentrations of Gbb-NBD (**1**) for 24h and (B) single-cell quantification of fluorescence intensity in (A). (Scale bar = 20 μm)

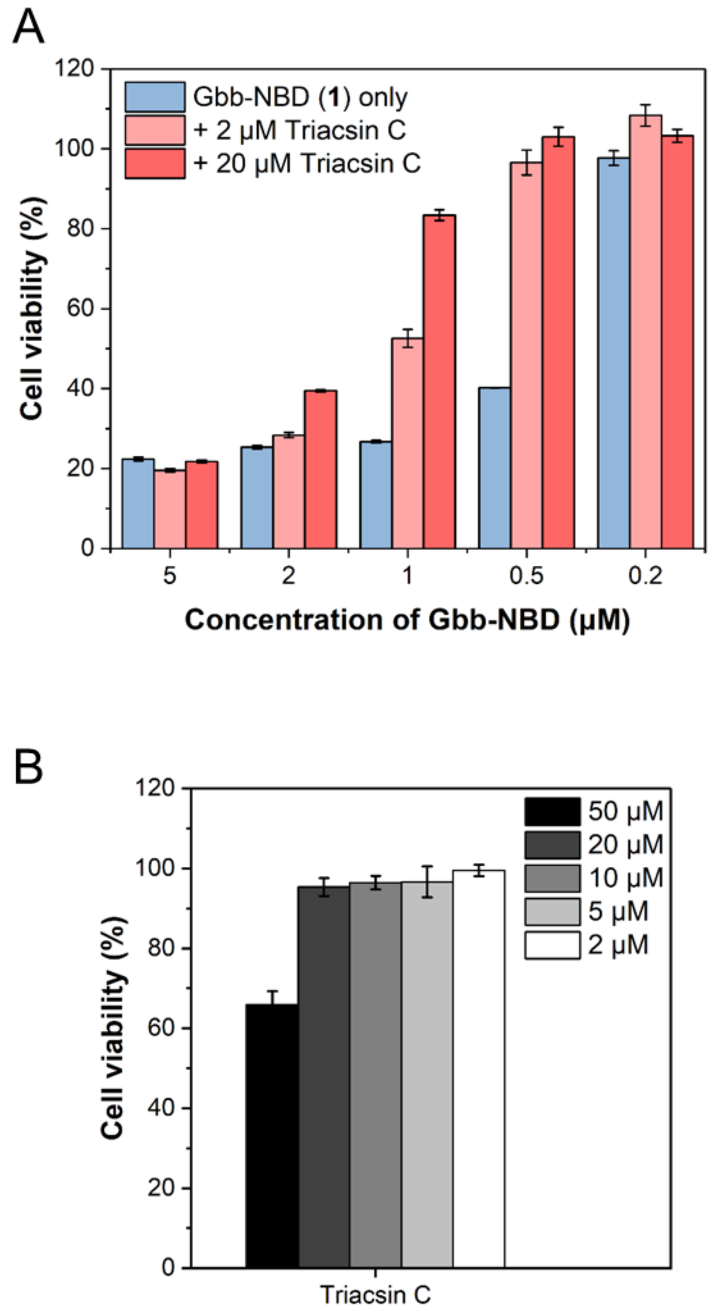

Figure S36. (A) Cell viability of HeLa cells treated with Gbb-NBD with or without the addition of Triacsin C for 24h. (B) Cell viability of HeLa cells treated with Triacsin C only.

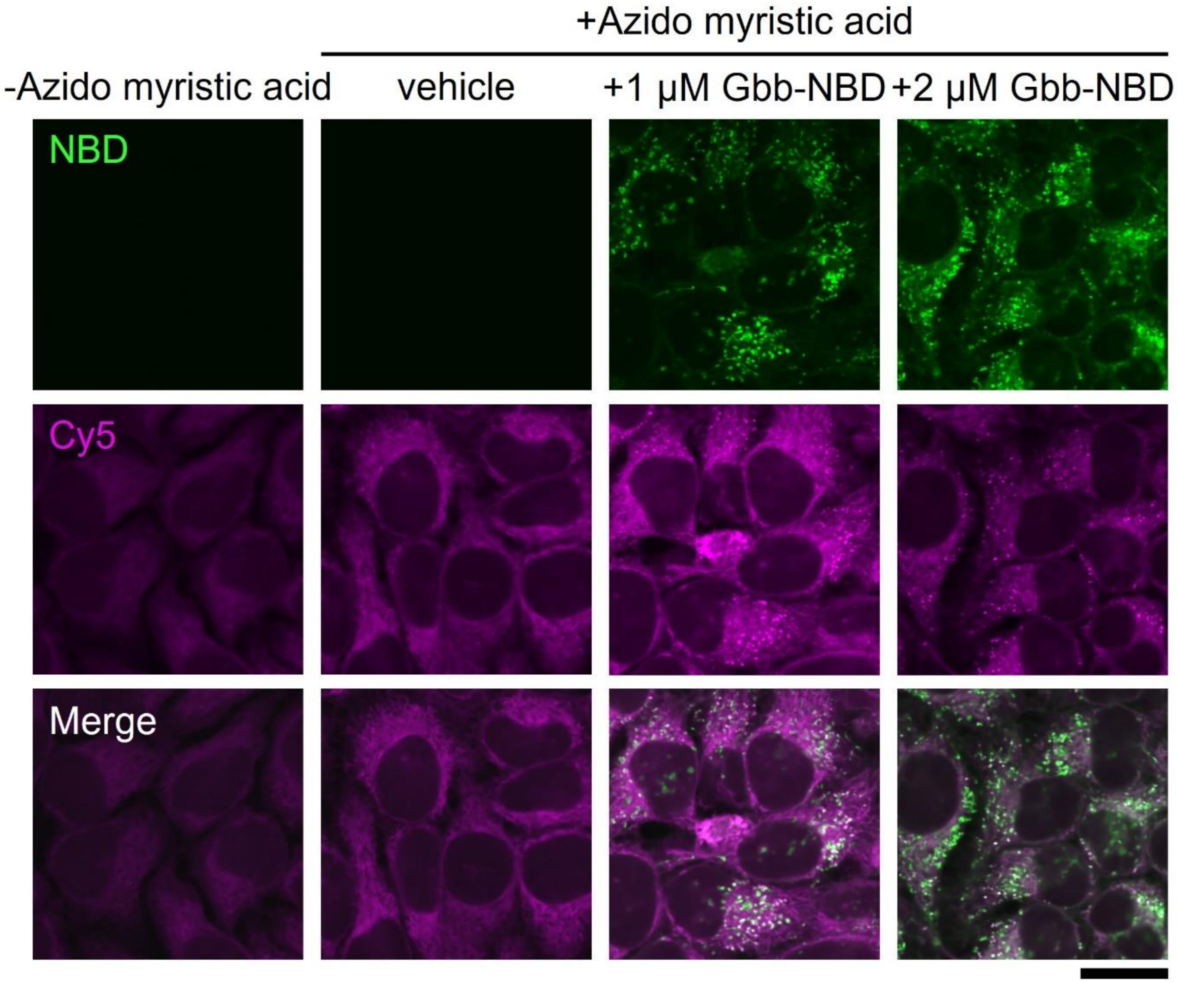

Figure S37. CLSM images of HeLa cells pretreated with azido myristic acid for 24h, then Gbb-NBD (**1**) was added to cells for another 24h compared to cells treated with azido myristic acid for 48h (vehicle) and blank (-azido myristic acid). (Scale bar = 20 μm)

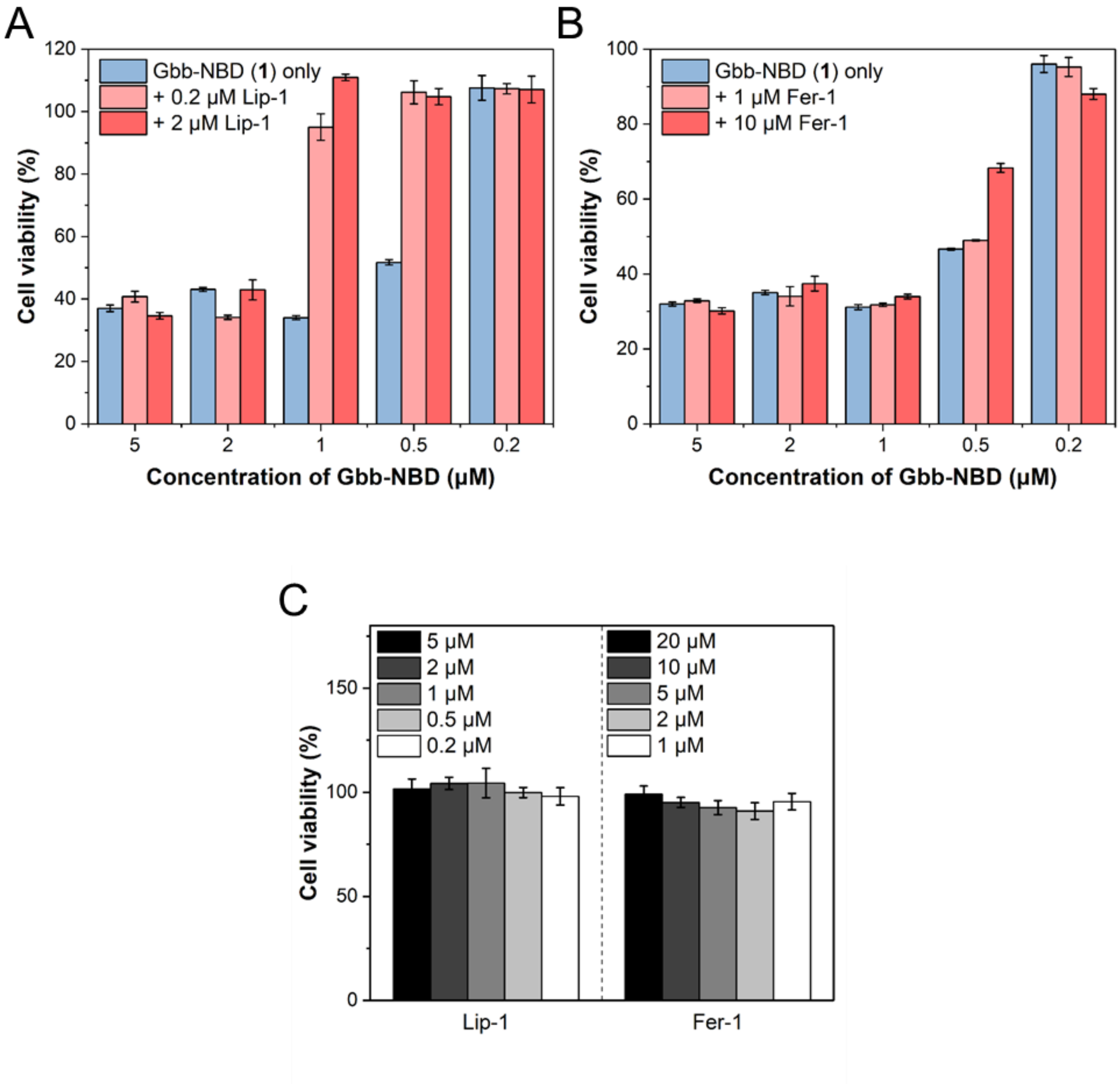

Figure S38. Cell viability of HeLa cells treated with Gbb-NBD (**1**) with or without the addition of (A) Lip-1 and (B) Fer-1 for 24h. (C) Cell viability of HeLa cells treated with Lip-1 and Fer-1 only for 24h.

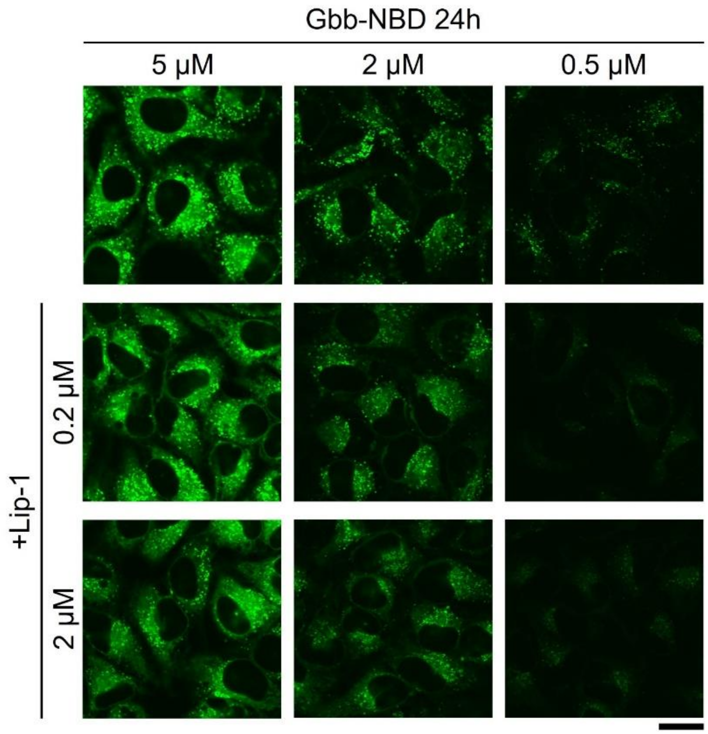

Figure S39. CLSM images of HeLa cells treated with different concentrations of Gbb-NBD (**1**) for 24h with or without Lip-1. (Scale bar = 20 μm)

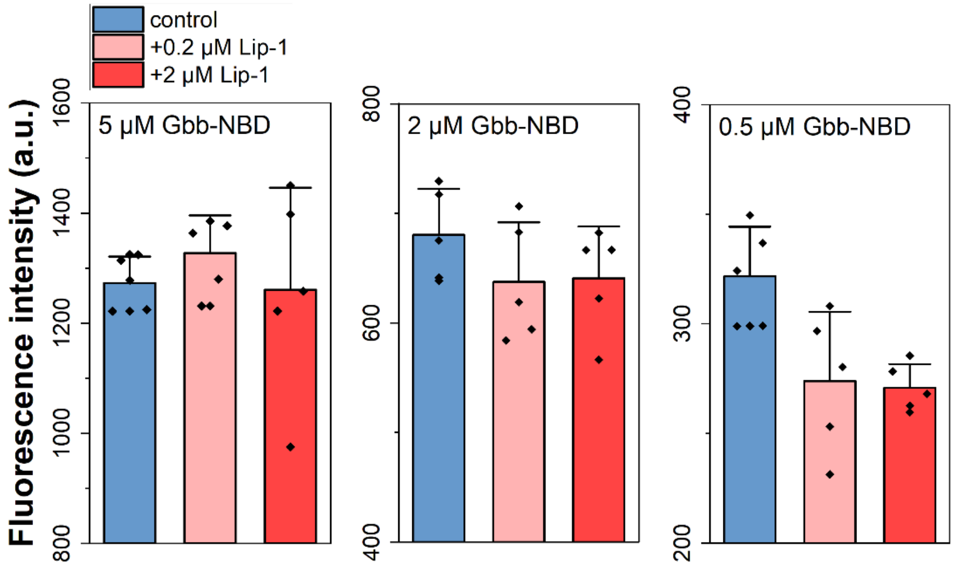

Figure S40. Single-cell quantification of fluorescence intensity in **Figure S39**.

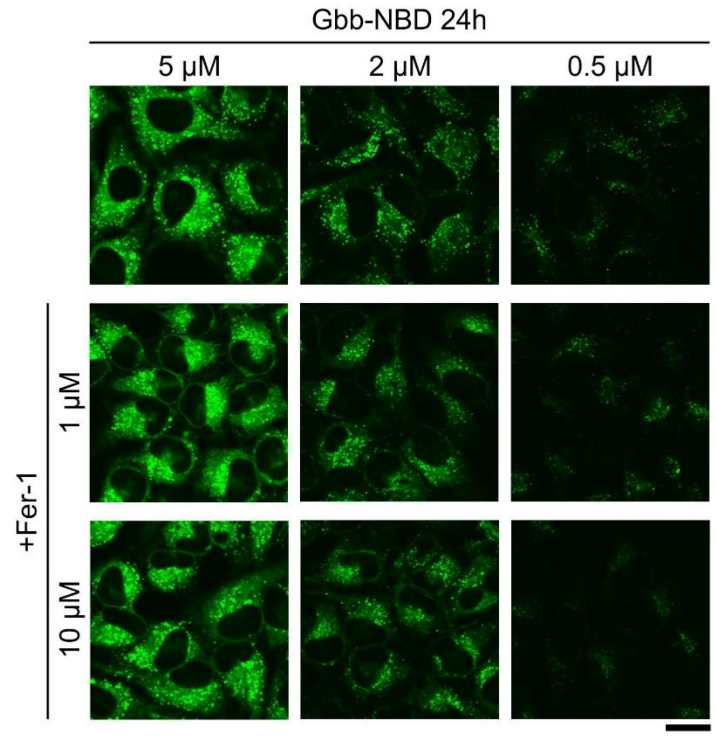

Figure S41. CLSM images of HeLa cells treated with different concentrations of Gbb-NBD (**1**) for 24h with or without Fer-1. (Scale bar = 20 μm)

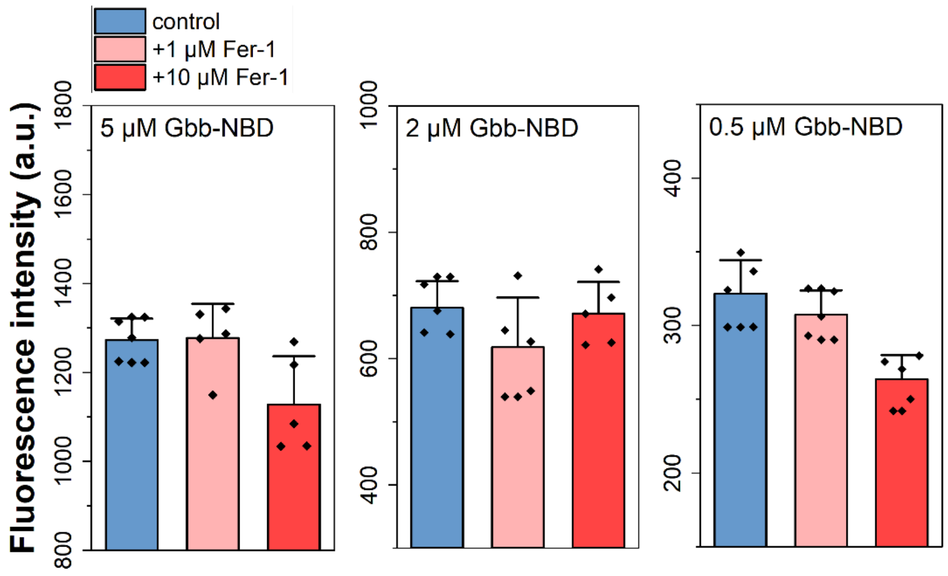

Figure S42. Single-cell quantification of fluorescence intensity in **Figure S41**.

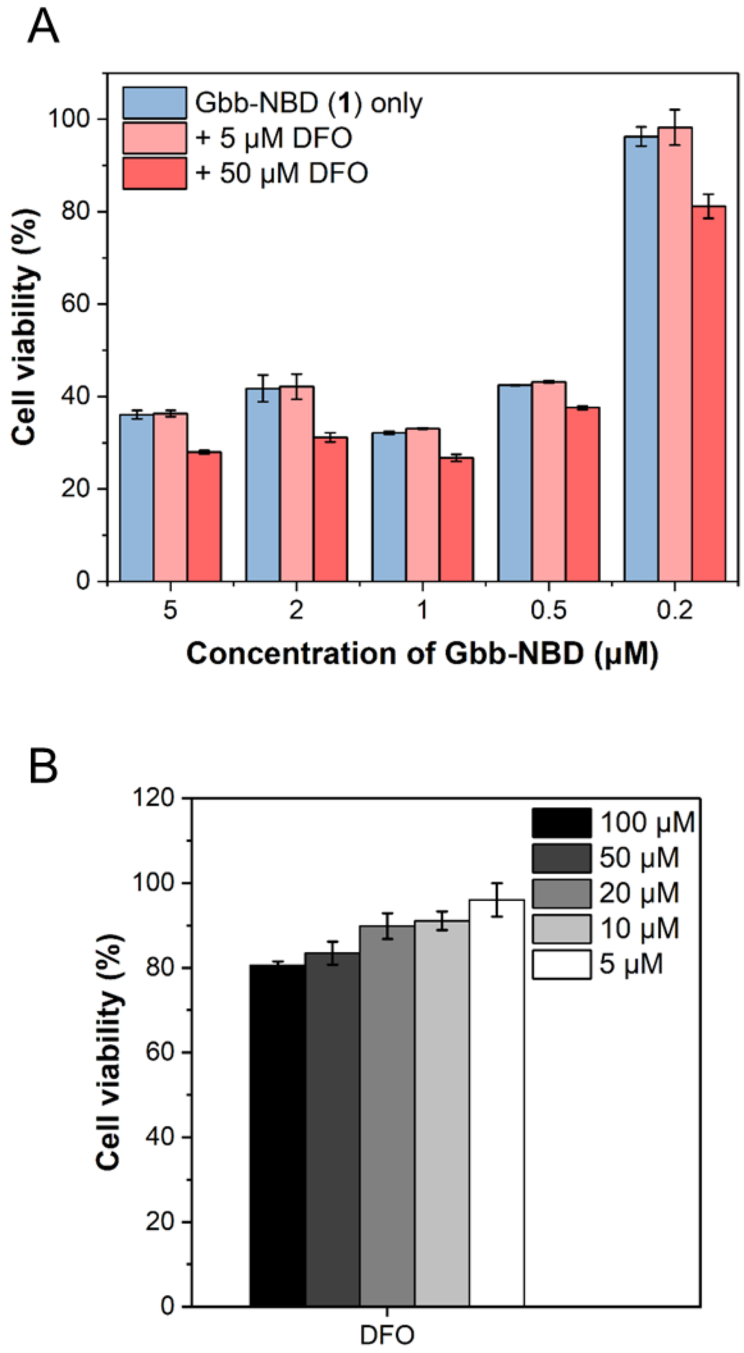

Figure S43. (A) Cell viability of HeLa cells treated with Gbb-NBD with or without the addition of DFO for 24h. (B) Cell viability of HeLa cells treated with DFO only for 24h.

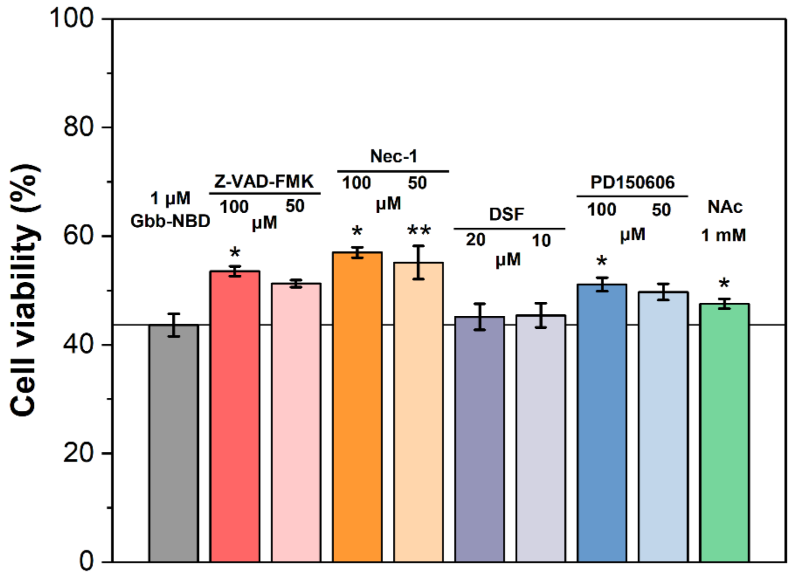

Figure S44. Cell viability of HeLa cells treated with 1 μM of Gbb-NBD with or without the addition of different cell death inhibitors for 24h.

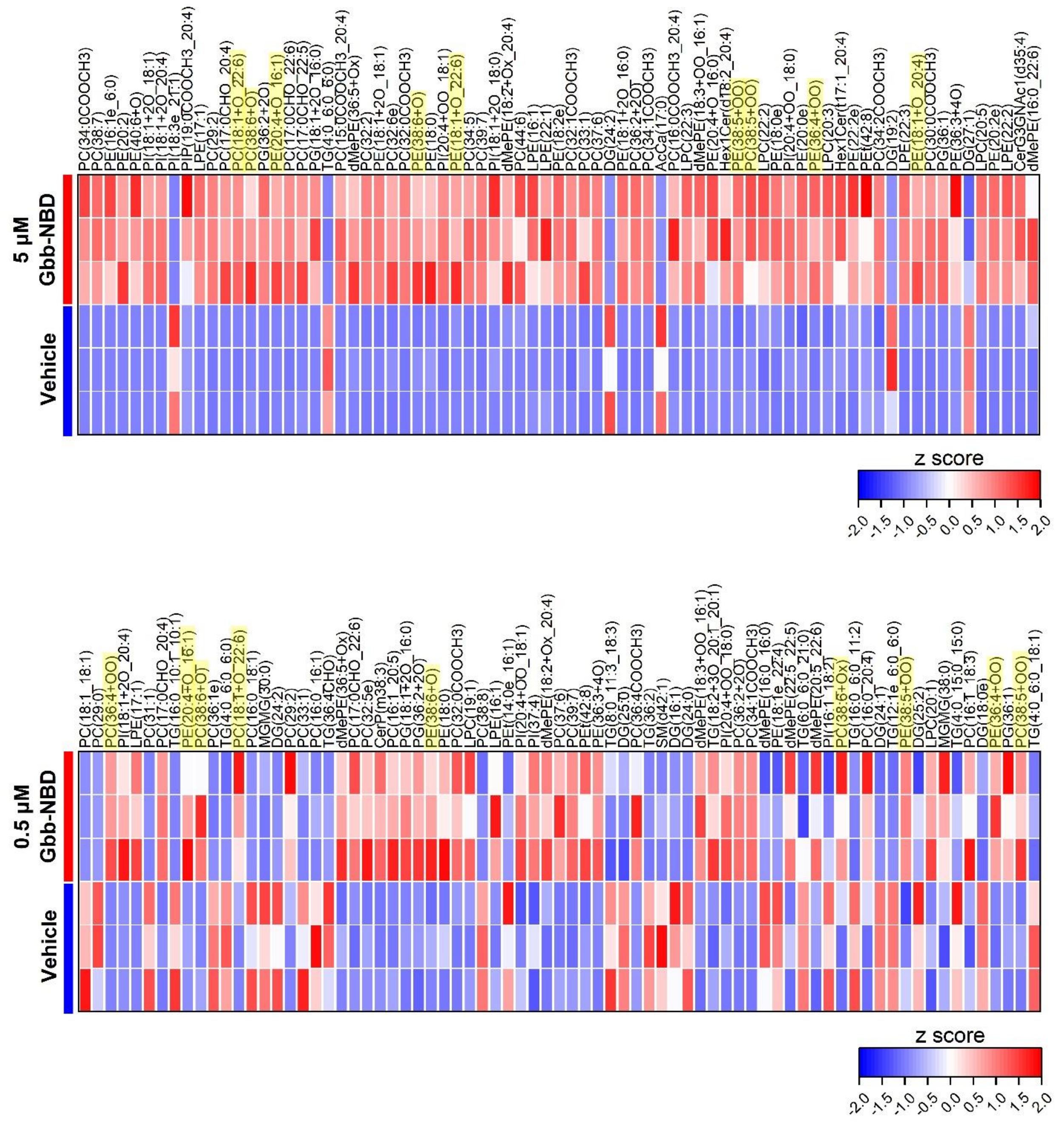

Figure S45. Heatmaps of top 75 significantly altered lipid species based on quantitative lipidomics of HeLa cells treated with **1** for 24h. Ferroptosis marker lipid species are highlighted.

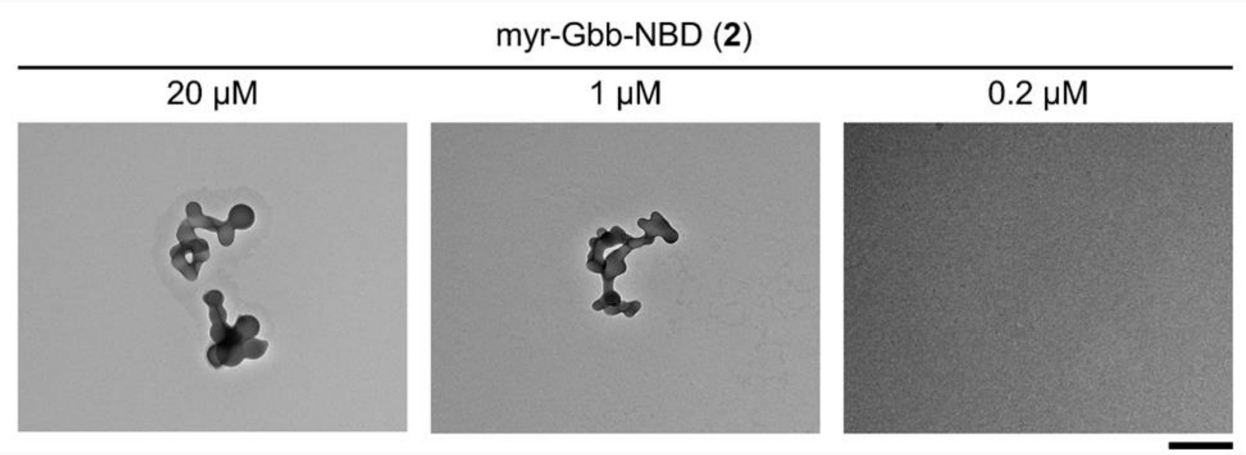

Figure S46. TEM images of 20, 1 and 0.2 μM of myr-Gbb-NBD (**2**) in ddH_2O at pH 7. (Scale bar = 400 nm)

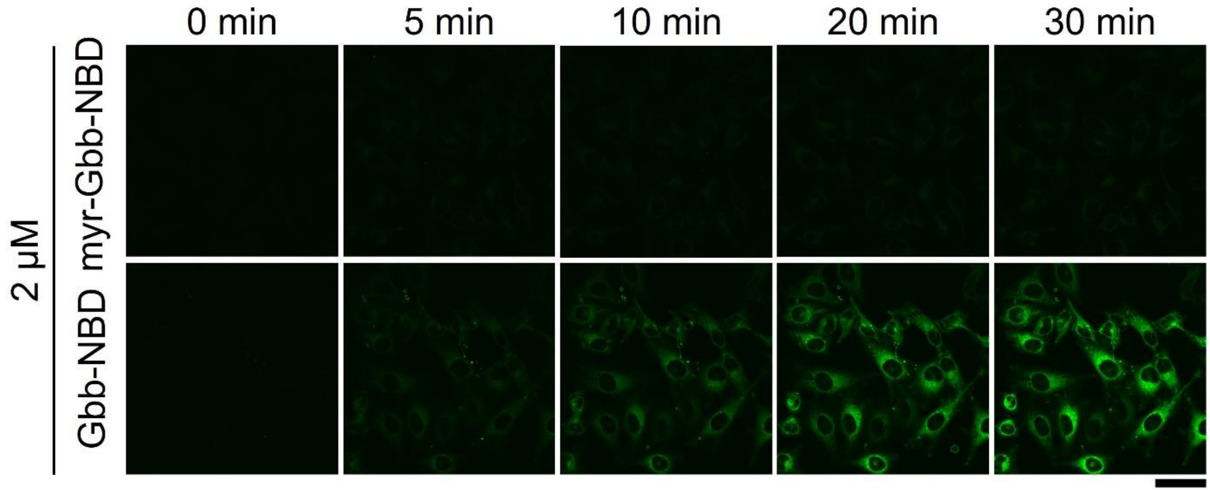

Figure S47. Time-lapse CLSM images of HeLa cells treated with 2 μM of myr-Gbb-NBD (**2**) or Gbb-NBD (**1**) (same-batch of cells). (Scale bar = 50 μm)

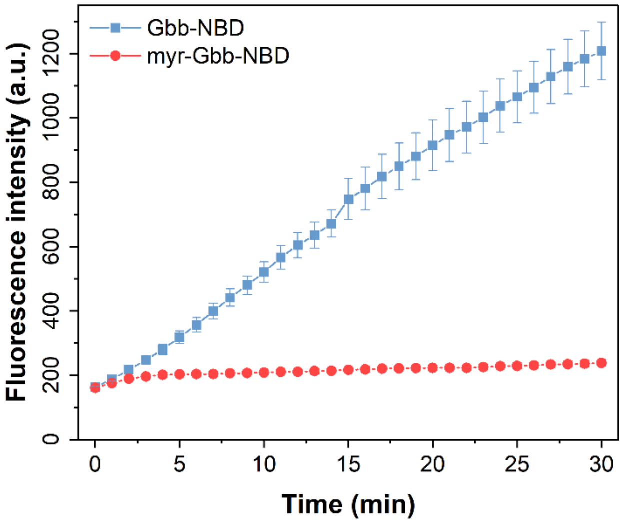

Figure S48. Single-cell quantification of fluorescence intensity in **Figure S47**.

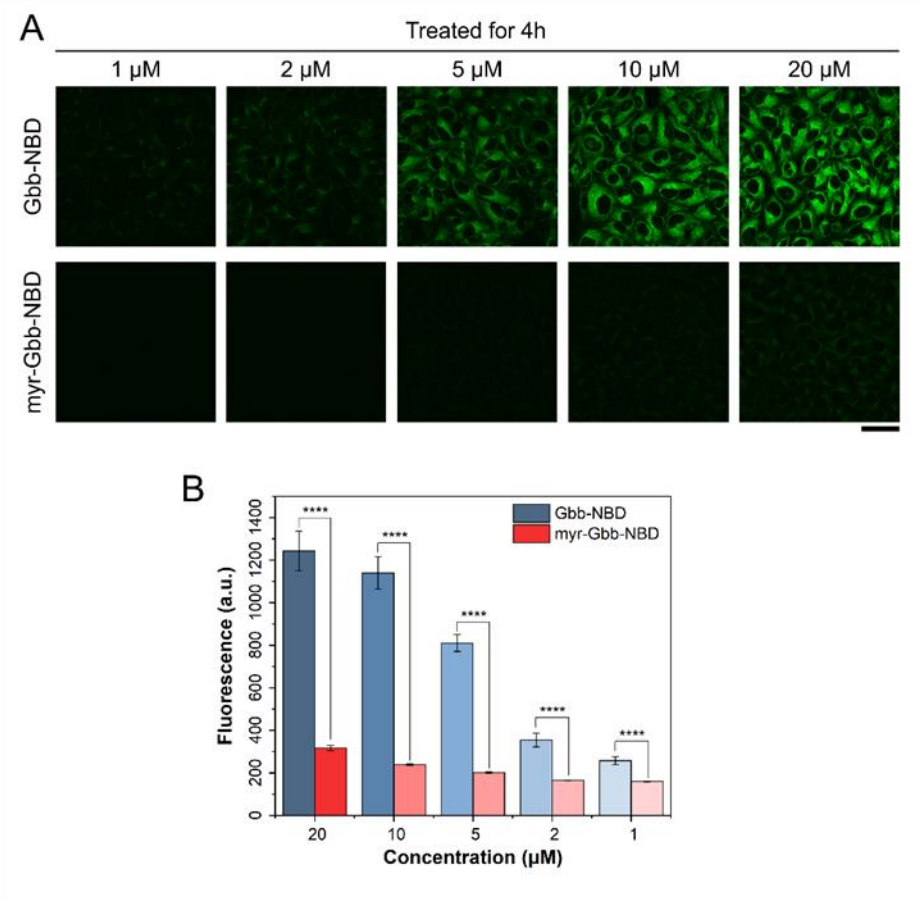

Figure S49. (A) CLSM images of HeLa cells treated with different concentrations of Gbb-NBD (**1**) and myr-Gbb-NBD (**2**) for 4h and (B) single-cell quantification of fluorescence intensity in (A). (Scale bar = 50 μm)

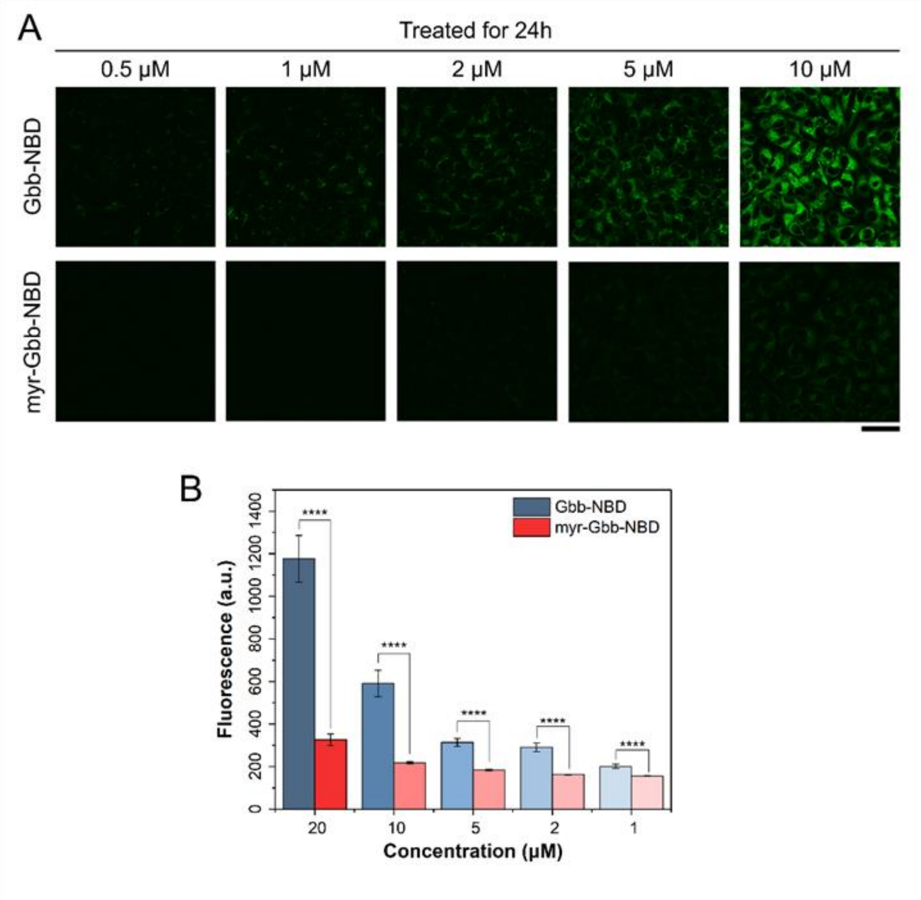

Figure S50. (A) CLSM images of HeLa cells treated with different concentrations of Gbb-NBD (**1**) and myr-Gbb-NBD (**2**) for 24h and (B) single-cell quantification of fluorescence intensity in (A). (Scale bar = 50 μm)

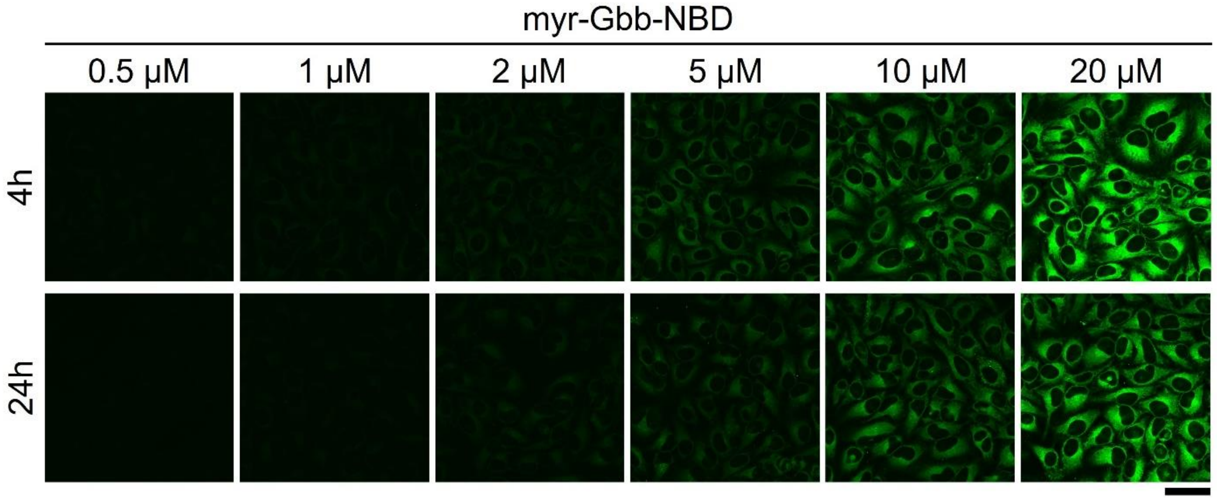

Figure S51. CLSM images of HeLa cells treated with different concentrations of myr-Gbb-NBD (**2**) for 4h and 24h. (Scale bar = 50 μm)

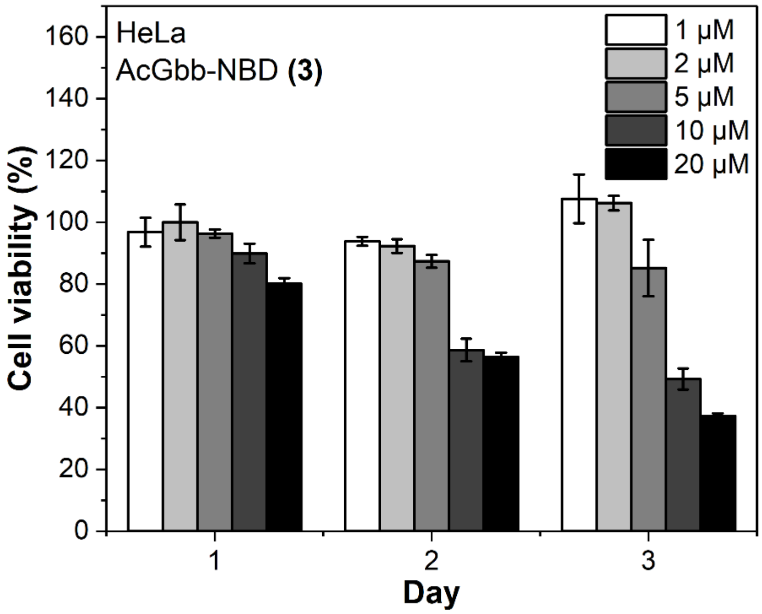

Figure S52. Cell viability of HeLa cells treated with AcGbb-NBD (**3**) for 1, 2 and 3 days.

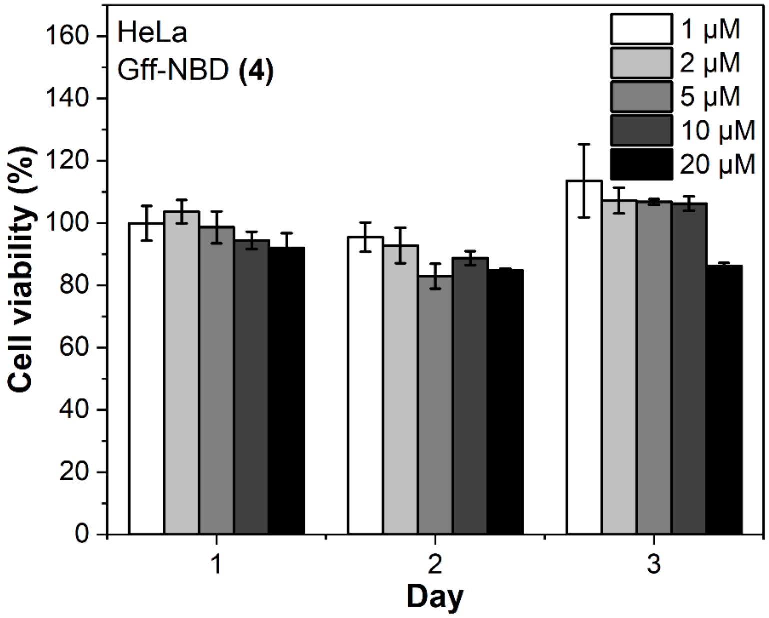

Figure S53. Cell viability of HeLa cells treated with Gff-NBD (**4**) for 1, 2 and 3 days.

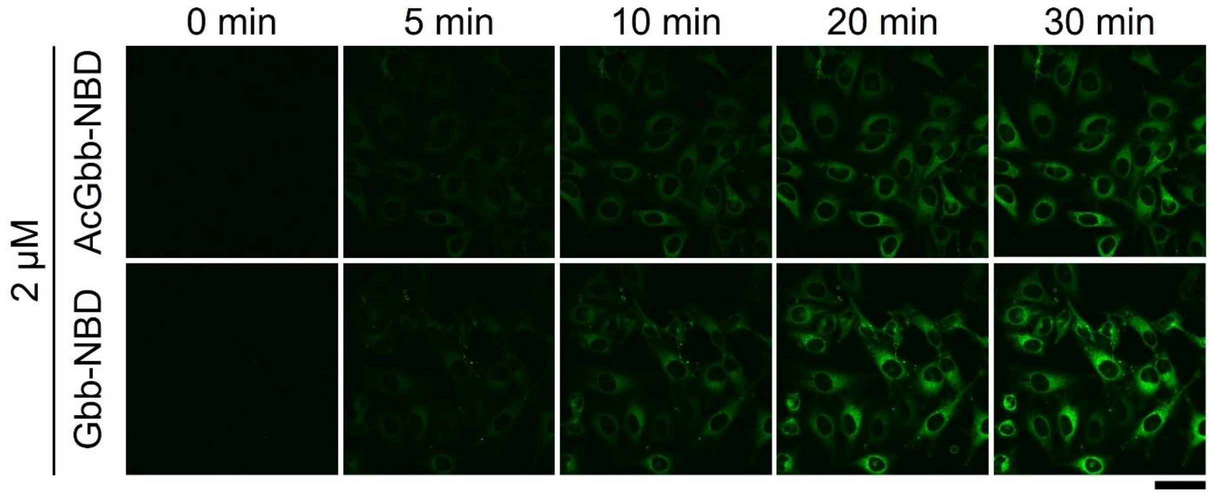

Figure S54. Time-lapse CLSM images of HeLa cells treated with 2 μM of AcGbb-NBD (**3**) or Gbb-NBD (**1**) (same-batch of cells). (Scale bar = 50 μm)

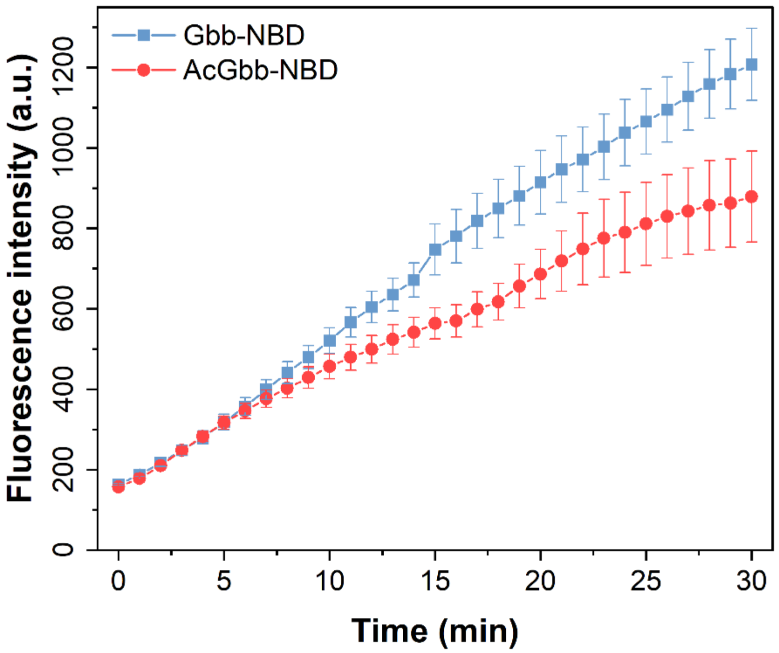

Figure S55. Single-cell quantification of fluorescence intensity in **Figure S54**.

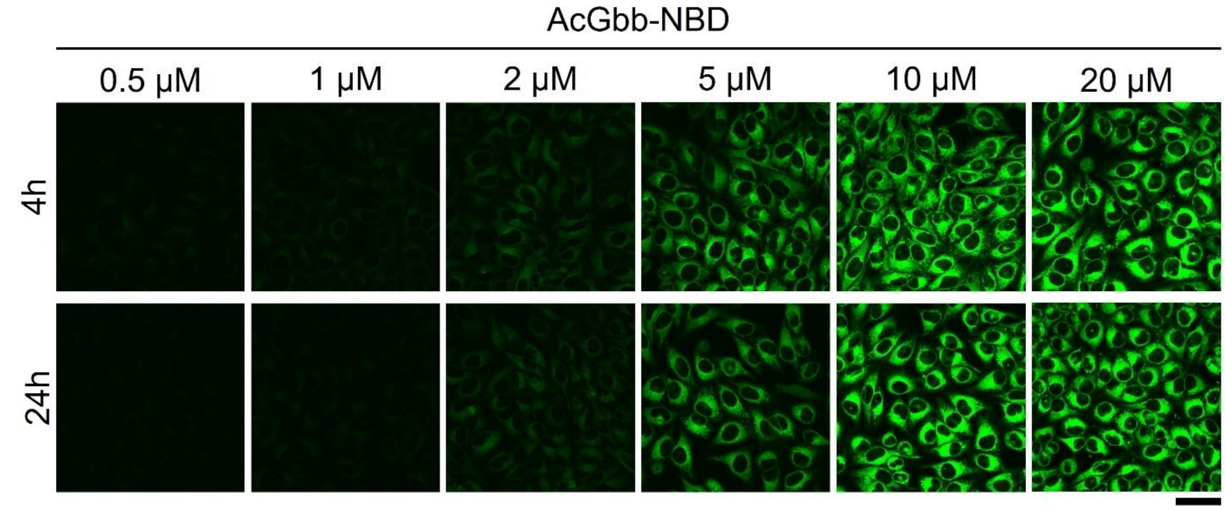

Figure S56. CLSM images of HeLa cells treated with different concentrations of AcGbb-NBD (**3**) for 4h and 24h. (Scale bar = 50 μm)

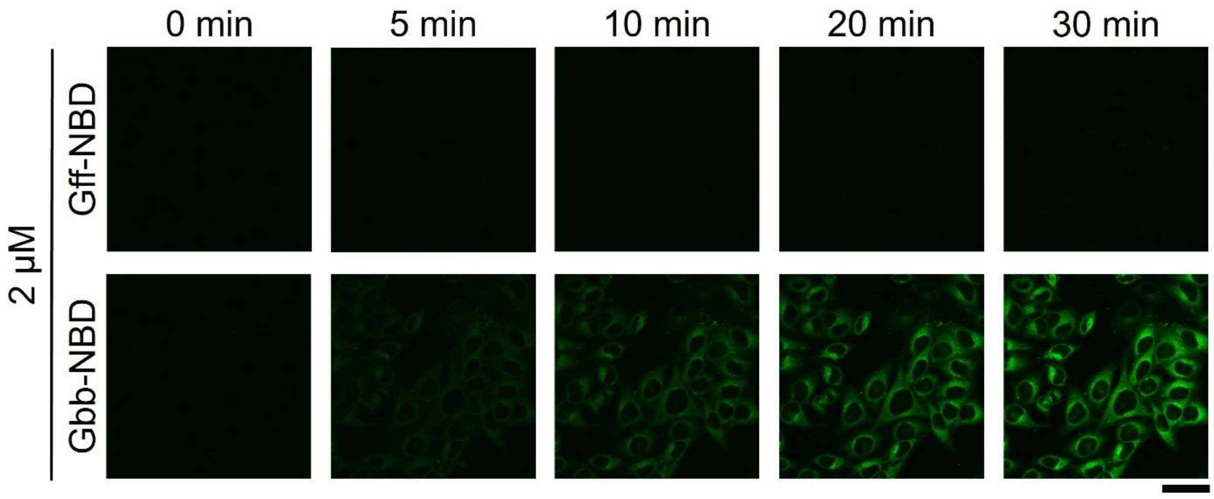

Figure S57. Time-lapse CLSM images of HeLa cells treated with 2 μM of Gff-NBD (**4**) or Gbb-NBD (**1**) (same-batch of cells). (Scale bar = 50 μm)

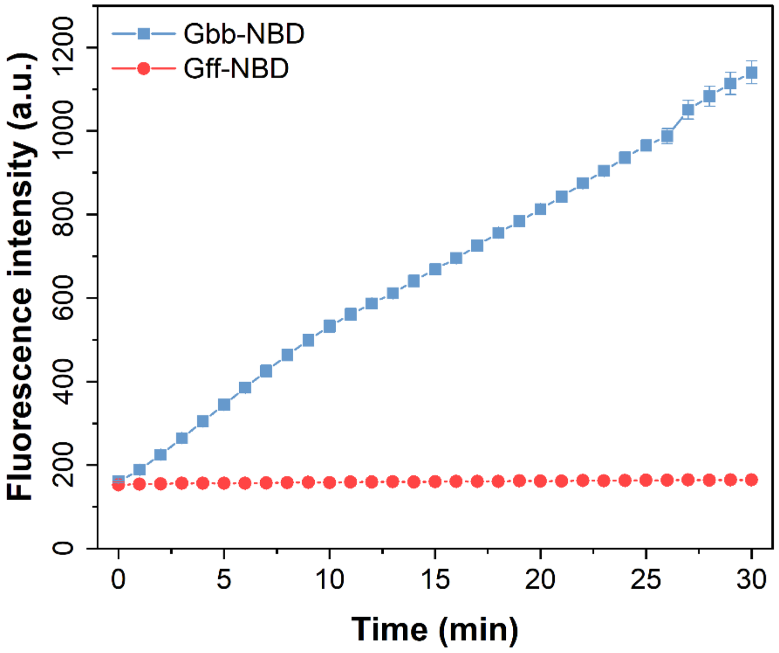

Figure S58. Single-cell quantification of fluorescence intensity in **Figure S57**.

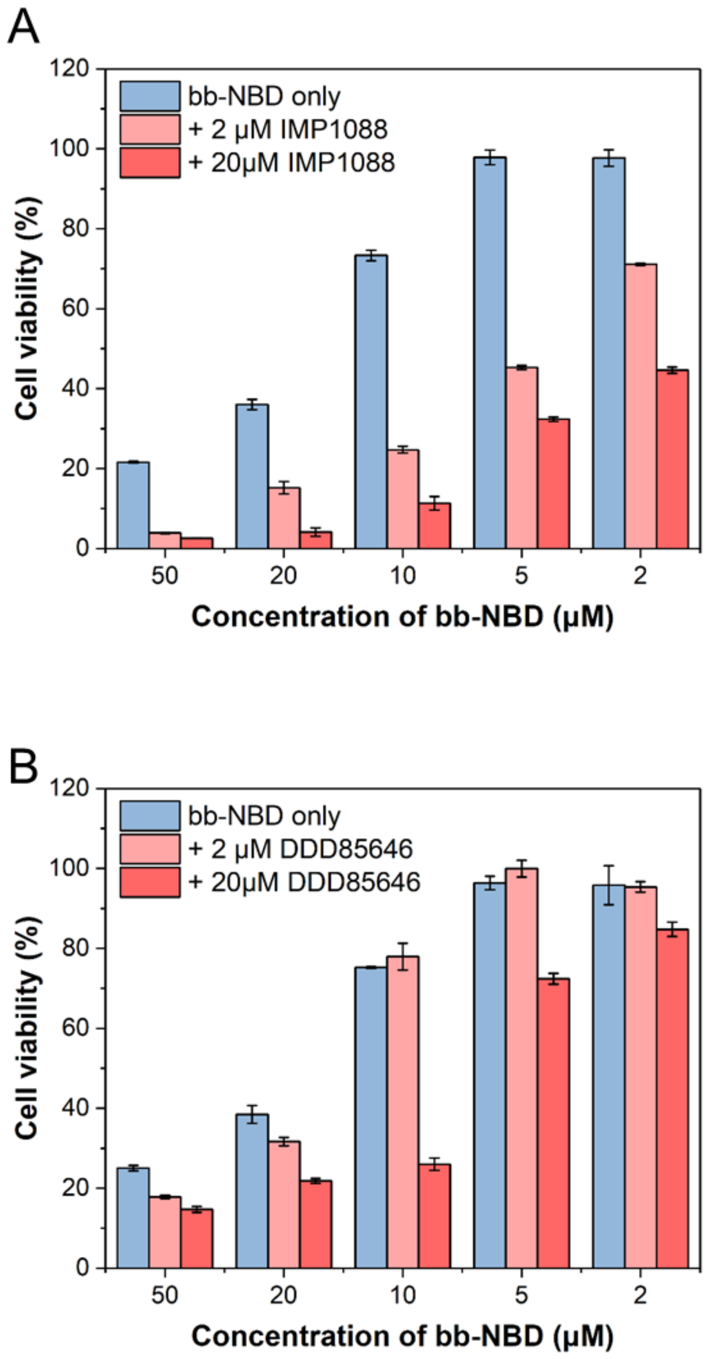

Figure S59. Cell viability of HeLa cells treated with bb-NBD with or without the addition of (A) IMP1088 and (B)DDD85646 for 24h.

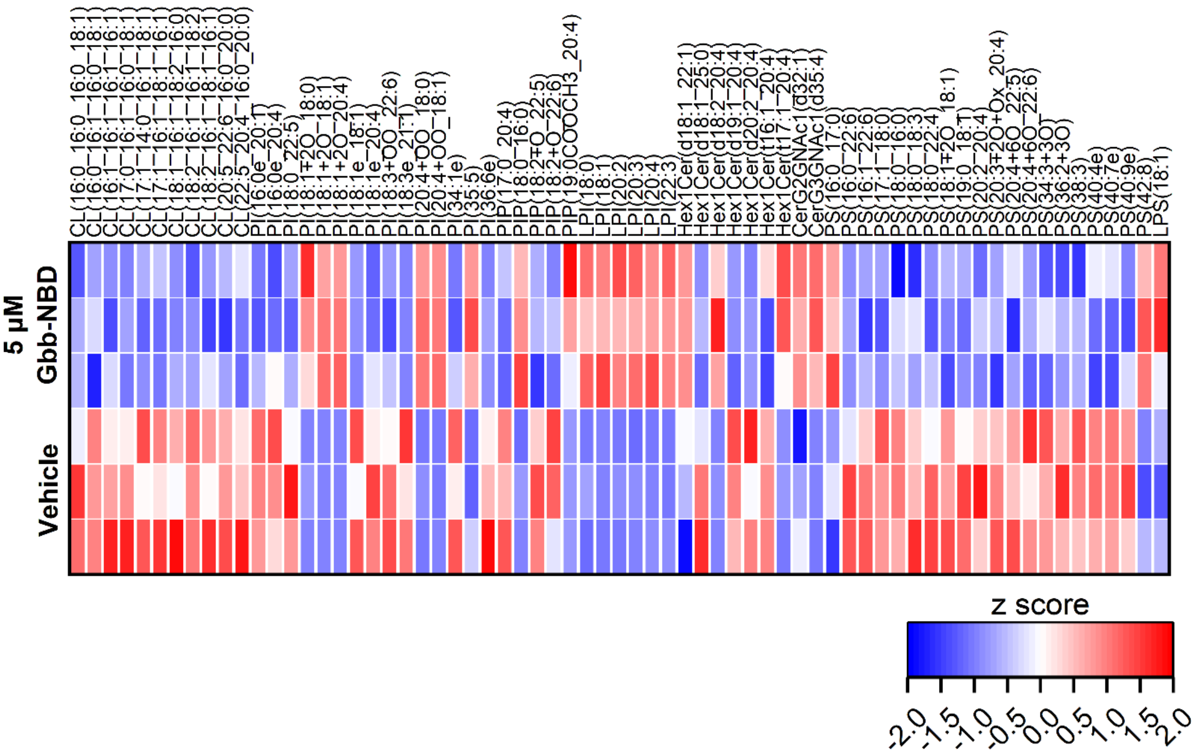

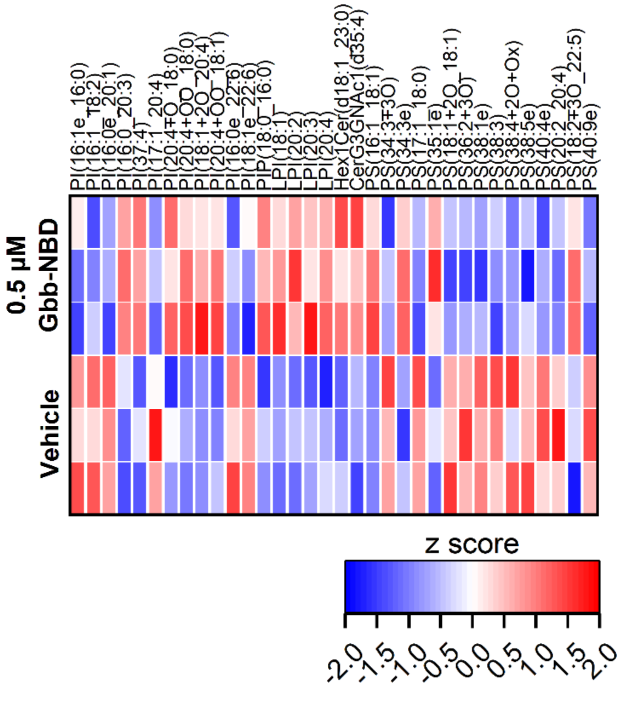

Figure S60. Heatmaps of other cell death related lipid species based on quantitative lipidomics of HeLa cells treated with Gbb-NBD (**1**) for 24h.

S14. Reference

(1) Breitkopf, S. B.; Ricoult, S. J. H.; Yuan, M.; Xu, Y.; Peake, D. A.; Manning, B. D.; Asara, J. M. A relative quantitative positive/negative ion switching method for untargeted lipidomics via high resolution LC-MS/MS from any biological source. *Metabolomics* **2017**, *13* (3), 30.
